# The CD123 antibody–drug conjugate pivekimab sunirine exerts profound activity in preclinical models of pediatric acute lymphoblastic leukemia

**DOI:** 10.1002/hem3.70063

**Published:** 2025-01-17

**Authors:** Ben Watts, Christopher M. Smith, Kathryn Evans, Andrew J. Gifford, Sara M. A. Mohamed, Stephen W. Erickson, Eric J. Earley, Steven Neuhauser, Timothy M. Stearns, Vivek M. Philip, Jeffrey H. Chuang, Patrick A. Zweidler‐McKay, Sribalaji Lakshmikanthan, Emily L. Jocoy, Carol J. Bult, Beverly A. Teicher, Malcolm A. Smith, Richard B. Lock

**Affiliations:** ^1^ Children's Cancer Institute, Lowy Cancer Research Centre, School of Clinical Medicine, UNSW Medicine & Health, UNSW Centre for Childhood Cancer Research UNSW Sydney Sydney New South Wales Australia; ^2^ Anatomical Pathology, NSW Health Pathology Prince of Wales Hospital Randwick New South Wales Australia; ^3^ School of Clinical Medicine, UNSW Medicine & Health UNSW Sydney Sydney New South Wales Australia; ^4^ Department of Pharmaceutics and Industrial Pharmacy Faculty of Pharmacy, Ain Shams University Cairo Egypt; ^5^ RTI International Research Triangle Park North Carolina USA; ^6^ The Jackson Laboratory for Mammalian Genetics Bar Harbor Maine USA; ^7^ The Jackson Laboratory for Genomic Medicine Farmington Connecticut USA; ^8^ ImmunoGen, Inc. Waltham Massachusetts USA; ^9^ The Jackson Laboratory Sacramento California USA; ^10^ National Cancer Institute Bethesda Maryland USA

## Abstract

Antibody–drug conjugates (ADCs) combining monoclonal antibodies with cytotoxic payloads are a rapidly emerging class of immune‐based therapeutics with the potential to improve the treatment of cancer, including children with relapse/refractory acute lymphoblastic leukemia (ALL). CD123, the α subunit of the interleukin‐3 receptor, is overexpressed in ALL and is a potential therapeutic target. Here, we show that pivekimab sunirine (PVEK), a recently developed ADC comprising the CD123‐targeting antibody, G4723A, and the cytotoxic payload, DGN549, was highly effective in vivo against a large panel of pediatric ALL patient‐derived xenograft (PDX) models (*n* = 39). PVEK administered once weekly for 3 weeks resulted in a median event‐free survival (EFS) of 57.2 days across all PDXs. CD123 mRNA and protein expression was significantly higher in B‐lineage (*n* = 65) compared with T‐lineage (*n* = 25) ALL PDXs (*p* < 0.0001), and mice engrafted with B‐lineage PDXs achieved significantly longer EFS than those engrafted with T‐lineage PDXs (*p* < 0.0001). PVEK treatment also resulted in significant clearance of human leukemia cells in hematolymphoid organs in mice engrafted with B‐ALL PDXs. Notably, our results showed no direct correlation between CD123 expression and mouse EFS, indicating that CD123 is necessary but not sufficient for in vivo PVEK activity. Importantly, a PDX with very high CD123 cell surface expression but resistant to in vivo PVEK treatment, failed to internalize the G4723A antibody while remaining sensitive to the PVEK payload, DGN549, suggesting a novel mechanism of resistance. In conclusion, PVEK was highly effective against a large panel of B‐ALL PDXs supporting its clinical translation for B‐lineage pediatric ALL.

## INTRODUCTION

Acute lymphoblastic leukemia (ALL) is the most common cancer in children and one of the leading causes of disease‐related mortality. In developed countries, 5‐year survival rates for ALL now exceed 90% for children and 75% for adolescents due to steady improvements in diagnosis, optimization of therapeutic regimens, and advances in supportive care over the past several decades.^1^ However, ALL is a heterogenous disease, and a subset of patients inevitably relapse and become refractory to standard‐of‐care therapy. Outcomes for patients with relapsed/refractory ALL remain extremely poor and modern treatment protocols designed to manage such cases often leave patients with long‐term health issues.^2^ Hence, there is a need to discover novel agents that are effective against relapsed/refractory ALL across different clinical subtypes, which minimize the adverse effects that patients experience.

In recent years, CD123 has emerged as a candidate for targeted therapy in hematological malignancies. CD123, encoded by the *IL3RA* gene, is the α subunit of the interleukin‐3 (IL‐3) receptor (IL‐3R). The IL‐3R has a high affinity toward the cytokine IL‐3, which upon binding induces tyrosine phosphorylation and activation of the JAK‐STAT pathway to regulate cell cycle progression, differentiation, and inhibition of apoptosis.^3^, ^4^ Early studies reported that CD123 was highly expressed in B‐ALL cells but low or absent in T‐ALL cells and normal lymphoid progenitors.^4^ Djokic et al. and Bras et al. both examined CD123 expression in subtypes of B‐ALL and found that CD123 was highest in patients with hyperdiploid karyotypes, while also increased in patients harboring translocations including *ETV6::RUNX1*, *BCR::ABL1*, *PBX1::TCF3*, and *MLL1* (*KMT2A*) rearrangements (MLLr‐ALL).^5^, ^6^ In another study by Angelova et al., high CD123 expression was more prevalent in Philadelphia Chromosome (Ph) positive patients harboring *BCR::ABL1* fusions, compared to Ph‐negative patients.^7^

An increasing number of antibody‐based targeted therapies including monospecific or bispecific antibodies and antibody–drug conjugates (ADCs) are under clinical evaluation for the treatment of cancers including leukemia.^8^ Pivekimab sunirine (PVEK, IMGN632) is an ADC comprising the CD123‐targeting antibody G4723A and the novel cytotoxic DNA alkylating payload, DGN549.^9^ Recently, several studies demonstrated that PVEK was highly effective against preclinical models of acute myeloid leukemia (AML) and blastic plasmacytoid dendritic cell neoplasm (BPDCN), and PVEK is currently being investigated in phase 1/2 clinical trials for both diseases (ClinicalTrials.gov Identifiers NCT03386513 and NCT04086264).^9^, ^10^, ^11^, ^12^ Specifically in ALL, activity has been shown in vitro against B‐cell ALL cell lines, however, there is a lack of studies investigating PVEK in vivo and the potential for this drug to treat pediatric ALL patients across different clinical subtypes has not been examined.^7^ To investigate PVEK for the treatment of pediatric ALL, we tested its efficacy against a panel of pediatric ALL patient‐derived xenograft (PDX) models representing a range of clinical subtypes and CD123 expression levels. Here, we demonstrate that PVEK was effective against most B‐ALL PDXs tested, including Ph^+^ and MLLr‐ALL subtypes, but less effective against T‐ALL PDXs. Our findings strongly support the potential for PVEK to be evaluated as a therapeutic option for relapsed/refractory pediatric B‐ALL patients.

## MATERIALS AND METHODS

### Establishment of PDXs

All animal experiments received prior approval from the Animal Care and Ethics Committee of UNSW Sydney (Sydney, NSW, Australia). The PDXs used throughout these experiments were previously established in 20–25 g female nonobese diabetic/severe combined immuno‐deficient (NOD.CB17‐*Prkdc* ^*scid*^/SzJ, NOD/SCID) or NOD/SCID/interleukin‐2 receptor γ–negative (NOD.Cg‐*Prkdc* ^*scid*^ *Il2rg* ^*tm1*^Wjl/SzJAusb, NSG) mice (Australian BioResources), as described previously.^13^, ^14^, ^15^ These PDXs are aggressive orthotopic models of the disease, exhibiting high‐level infiltration of bone marrow (BM) and spleen, and disseminating to other organs and the peripheral blood (PB). Demographic and molecular characteristics of the 40 pediatric ALL PDXs used in this study are included in Supporting Information S1: Table S1.

### Quantification of CD123 messenger RNA (mRNA) expression in ALL subtypes

CD123 (*IL3RA*) mRNA expression was assessed in 90 pediatric ALL PDXs via whole‐transcriptome RNA sequencing (RNA‐seq) as described previously.^15^ RNA‐seq mRNA expression values are expressed as fragments per kilobase per million reads (FPKM). Raw gene counts were used as an input to the B‐ALL prediction tool ALLSorts to subclassify the 65 B‐lineage PDXs.^16^ The selection of PDXs for in vivo experiments was based on CD123 mRNA expression.

### Quantification of CD123 cell surface expression

CD123 cell surface expression was measured by flow cytometry. Briefly, PDX cells were incubated with either the anti‐CD123 antibody clone 9F5 (catalog number #555642; BD Biosciences) or an isotype control, mouse IgG1, κ (catalog number #555746; BD Biosciences), and labeled with FITC Goat Anti‐Mouse Ig (catalog number #554001; BD Biosciences) according to the manufacturer's recommendation. The mean fluorescence intensity (MFI) was determined from the peak fluorescence channel geometric mean and relative fluorescence intensity (RFI) was defined as the ratio of MFIs for the CD123 antigen and isotype control.

### PVEK tolerability testing in NSG mice

PVEK and the control ADC were provided by ImmunoGen, Inc. and formulated in 10 mM succinate, 8% trehalose, 50 µM sodium bisulfite, 0.01% polysorbate‐20 (pH 4.2) or 10 mM succinate, 8.5% trehalose, and 50 µM sodium bisulfite (pH 4.2), respectively. PVEK is a cysteine‐linked conjugate of the G4723A antibody and DGN549 payload, at a drug‐to‐antibody ratio (DAR) of 1.7–1.9. The control ADC was a conjugate of the huKTIc442 CysMab antibody (an antibody that binds to Kunitz soybean trypsin inhibitor) and the DGN549 payload, at a DAR of 1.87.^17^ Tolerability testing was undertaken in naïve female NSG mice aged 10–12 weeks. Mice were treated with the company‐recommended dose of PVEK (0.24 mg/kg), or with 75% (0.18 mg/kg), 50% (0.12 mg/kg), and 25% (0.06 mg/kg) of the recommended dose, alongside a vehicle control and control ADC treated group, in an unblinded fashion. Mice were treated once per week for three weeks via intravenous (IV) injection at a maximum administered volume of 5 µL/g body weight. Mice were treated with the platelet‐activating factor (PAF) receptor antagonist CV‐6209 (Cayman Chemical) at 5 mg/kg in normal saline (0.9% sodium chloride) 1 h before antibody treatment to prevent potential antibody‐associated hypersensitivity reactions. In the second tolerability study, mice were also treated with an FcR blocker (chimeric KTI, 400 mg/kg) 24 h before each PVEK treatment, formulated in 50 mM potassium phosphate, 50 mM potassium chloride, and 2 mM EDTA and administered via intraperitoneal (IP) injection, to prevent non‐specific binding of PVEK. PB was collected and analyzed for hematology and biochemistry parameters. Mice were also weighed no less than weekly to monitor any treatment‐induced weight loss.

### Assessment of PVEK efficacy in vivo

Efficacy studies were initiated in female NSG mice that were 6 to 8 weeks old. Four different formats of efficacy studies were undertaken: (1) conventional (eight mice/treatment group/PDX); (2) dose‐escalation (eight mice/treatment group/PDX); (3) single‐mouse trial (SMT, one mouse/PDX); and (4) timepoint study (three mice/treatment group/PDX).^18^ A cohort size of eight mice was determined for the conventional and dose‐escalation studies as this is sensitive enough to detect a twofold difference in event‐free survival (EFS) between control and treated mice at a significance level of *p* < 0.05 and power of 0.8.^19^ In each study, mice were inoculated with PDX cells (2–5 × 10^6^ cells per mouse) via the lateral tail vein and engraftment was assessed by weekly flow cytometric enumeration of the proportion of human CD45^+^ (%huCD45^+^) cells versus total CD45^+^ cells (human and murine) in the PB. Treatment began once the median %huCD45^+^ cells in the PB of each cohort of mice was at least 1%, or in the case of the SMT study, when the individual mouse reached at least 1% huCD45^+^ cells in the PB. As in the tolerability study, mice were treated with an FcR blocker (chimeric KTI, 400 mg/kg) 24 h before each PVEK treatment and CV‐6209 one hour before the PVEK treatment. PVEK was administered via IV injection once per week for 3 weeks at 0.24 mg/kg, except in the dose‐escalation study, where it was also administered at 0.06 and 0.12 mg/kg. The conventional efficacy study also included a group treated with the same control ADC as in the tolerability study (0.24 mg/kg IV weekly × 3), while the conventional and dose‐escalation studies also included control vehicle‐treated groups. All doses of PVEK used in this study were equivalent to those achievable in humans.

Events were predefined as 25% huCD45^+^ in the PB, or mice exhibiting signs of leukemia‐related morbidity with at least 50% huCD45^+^ infiltration in two or more major organs, as assessed by flow cytometry. EFS was determined in the conventional, dose‐escalation, and SMT studies, and was calculated as the number of days from treatment initiation to event or until leukemia‐related morbidity. The exact time‐to‐event was estimated by interpolating between the measurements directly preceding and following the event, assuming log‐linear growth, and represented using Kaplan–Meier analysis.^20^ In the timepoint study, organ infiltration was examined via flow cytometry analysis of the %huCD45^+^ in cardiac puncture, spleen, and BM samples obtained on Day 0 or Day 28/event posttreatment initiation. To morphologically assess tumor burden, spleen, and femur samples were fixed in 10% neutral buffered formalin for 24 h, following which femur samples required decalcification for 48 h in 0.39 M EDTA and 0.36 M sodium hydroxide. Samples were then routinely processed, embedded in paraffin for sectioning, and sections stained with hematoxylin and eosin (H&E) and assessed by a practicing pediatric pathologist (AJG) using a standard light microscope. The spleen and femur samples were assessed blinded to the treatment group. Representative images were captured at ×600 magnification using an Olympus BX53 light microscope and CD73 camera with CellSens software. Response to treatment was measured using multiple methods including (1) EFS or differences between median EFS in drug‐treated (T) and vehicle control (C) groups (T‐C and T/C); (2) maximum decrease of %huCD45^+^ from baseline following treatment initiation; (3) area over the curve (AOC), determined by analysis of %huCD45^+^ with respect to time; and (4) objective response measures (ORM) based on stringent clinical criteria assessed at Day 42 posttreatment initiation (see Supporting Information Methods).^21^

### Ex vivo cytotoxicity assay

ALL PDXs were seeded at their optimal seeding density (Supporting Information S1: Table S2) in QBSF‐60 medium (Quality Biological Inc.) and equilibrated for 3 h at 37°C, 5% CO_2_ before treatment. Cells were treated with DGN549 or a vehicle control of ethanol at a range of concentrations (0.1 pM to 100 µM) for 48 h. Viability after 48 h of drug exposure was determined using the AlamarBlue reduction assay and fluorescence was measured using a Victor X3 Multilabel Plate Reader (560 nm excitation and 590 nm emission; PerkinElmer). IC_50_ values were calculated by nonlinear regression analysis using GraphPad Prism (version 8.4.1).

### Assessment of G4723A antibody internalization ex vivo

Internalization of the CD123‐targeting antibody G4723A (catalog number #624255; BD Biosciences) was assessed with an indirect immunofluorescence assay. Briefly, 5 × 10^5^ PDX cells were incubated with 100 nM G4723A at 37°C for 1 h. The cells were then washed with phosphate buffer saline (PBS) and fixed with 4% paraformaldehyde (PFA) in FluoroDish™ (World Precision Instruments Inc.). Subsequently, the cells were stained with Hoechst 33 342 (ThermoFisher) to visualize the nuclei and AF™ 647‐labeled anti‐human secondary antibody (ThermoFisher) to visualize the G4723A antibody. *Z*‐stacks were captured in an unblinded fashion using a Leica TCS SP8 DLS (Leica Microsystems) at 400x magnification with the ×40 oil immersion objective. The images were normalized to control samples stained with the secondary antibody alone. ImageJ software was used to generate plot profiles of AF™ 647 and Hoechst 33342, representing G4723A and the nuclei, respectively. Internalization of G4723A was quantified by recording the maximum gray values of AF™ 647 that overlap with the gray values of Hoechst 33342 in at least 80 cells for each PDX.

### Statistical analysis

For the comparison between B‐ and T‐cell lineage PDXs, differences in CD123 expression and EFS were determined using the Wilcoxon Rank Sum test. The correlation between CD123 mRNA expression and RFI was calculated using Pearson's correlation. Comparison of EFS between treatment groups was tested using the G^ρ^ test of Harrington & Fleming (*α* = 0.05, two‐sided alternative) with *ρ* = 1, which is equivalent to the Peto & Peto modification of Gehan‐Wilcoxon.^22^ For the SMT study, all correlations between expression (mRNA or RFI) and efficacy (EFS or ORM) were calculated using Pearson's correlation. For the assessment of G4723 antibody internalization ex vivo, the median maximum overlaying gray values of cells were compared between PDXs using the Wilcoxon Rank Sum test. Unless otherwise stated above, experimenters were not blinded to group assignments.

## RESULTS

### CD123 mRNA and cell surface expression in pediatric ALL PDXs

**Figure 1:**
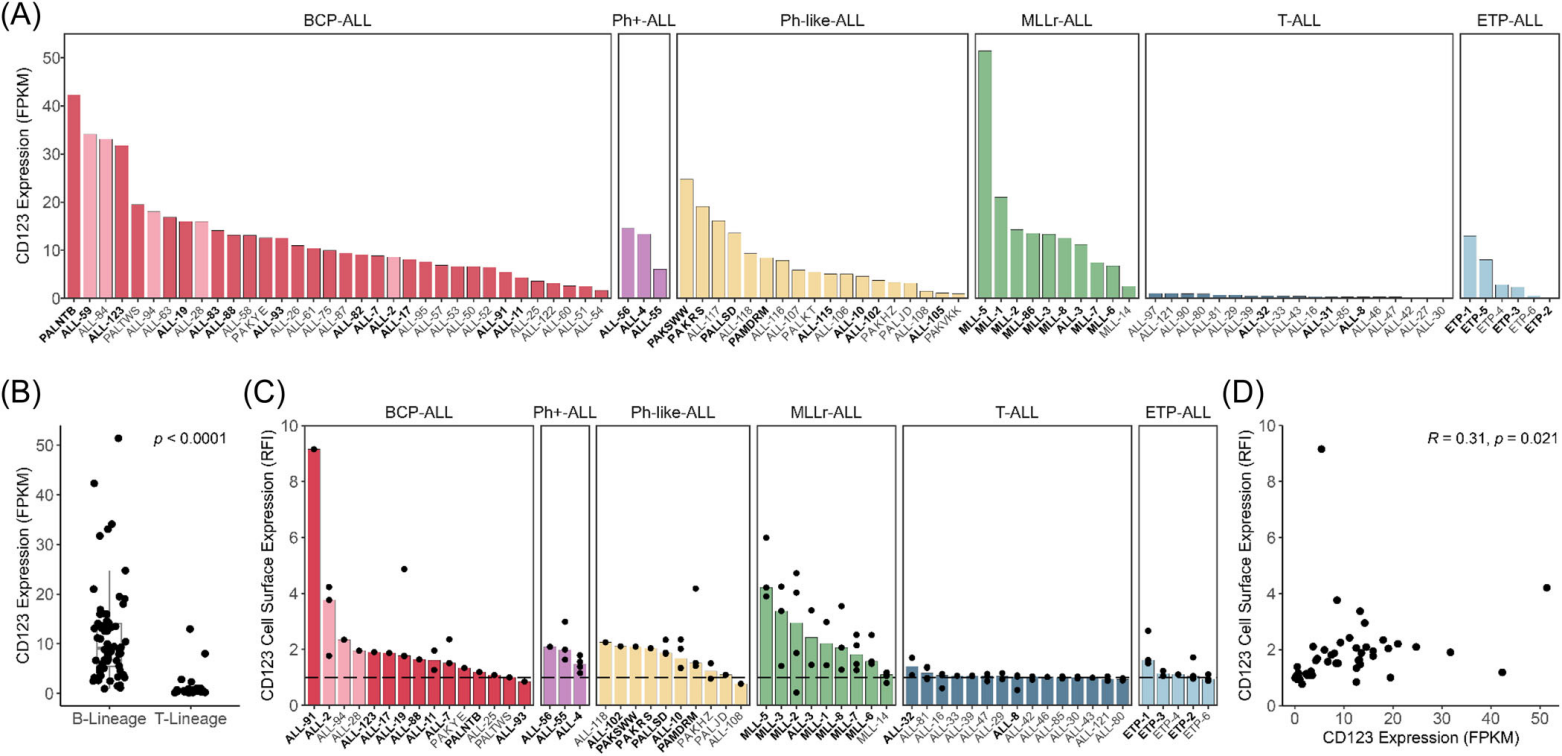
**CD123 mRNA and cell surface expression in the panel of pediatric ALL PDXs used in this study**. **(A)** CD123 (*IL3RA*) mRNA expression across 90 PDXs (*n* = 1), displayed by subtype. PDXs selected for the SMT are marked in bold. **(B)** CD123 mRNA expression stratified according to B‐lineage versus T‐lineage ALL PDXs, compared using the Wilcoxon Rank Sum test. **(C)** CD123 RFI in a panel of 57 ALL PDXs, displayed by subtype. Dots indicate the RFI of each biological replicate (number of replicates ranged from 1 to 7) and bars indicate the median RFI for each PDX. The dashed line indicates an RFI of 1, equivalent to no expression. PDXs selected for the SMT are marked in bold. **(D)** Correlation between CD123 mRNA expression and CD123 RFI. The pink columns in A and C denote hyperdiploid BCP‐ALL PDXs, as defined by ALLSorts.^16^ ALL, acute lymphoblastic leukemia; mRNA, messenger RNA; *P*, *p*‐value; *R*, Pearson's correlation coefficient; RFI, relative fluorescence intensity.

The molecular characterization (RNA‐seq, whole‐exome sequencing, DNA copy number analysis) of 90 pediatric ALL PDXs has been previously reported by our group.^15^ These 90 PDXs encompass six ALL subtypes broadly representing B‐cell lineage (including B‐cell precursor [BCP‐ALL], Ph‐positive [Ph^+^‐ALL], Ph‐like [Ph‐like‐ALL], and MLLr‐ALL) and T‐cell lineage (including T‐cell [T‐ALL] and early T‐cell precursor [ETP‐ALL] ALL. CD123 mRNA expression varied across the ALL PDXs, ranging from 0.1 to 51.4 FPKM (Figure 1A). The median FPKM of CD123 was 18.6‐fold higher in B‐cell lineage PDXs compared to T‐cell lineage PDXs (*p* < 0.0001) (Figure 1B). Cell surface expression of CD123 ranged from 0.7 to 9.2 RFI (Figure 1C). When the 65 B‐lineage PDXs were subclassified using the ALLSorts prediction tool, three out of the highest four CD123 cell surface expressing BCP‐ALL PDXs were predicted as hyperdiploid (ALL‐2, ALL‐28, ALL‐94).^16^ Furthermore, CD123 cell surface expression (RFI) measured across a subset of PDXs had a weak positive correlation with mRNA expression (Pearson's *R* = 0.31, *p* = 0.021) (Figure 1D).

### In vivo efficacy of PVEK against pediatric ALL PDXs

To identify a suitable dose for evaluating PVEK efficacy in vivo, a tolerability study was conducted in naïve NSG mice. PVEK was administered once a week for 3 weeks at 4 doses (0.06, 0.12, 0.18, and 0.24 mg/kg). PVEK was generally well tolerated at all doses tested, however, one mouse from each of the two highest dosing groups (0.18 and 0.24 mg/kg) was lost due to significant weight loss (at least 20% weight loss from treatment initiation) or diarrhea (Supporting Information S1: Figure S1A). No other mice exhibited signs of severe weight loss or acute toxicity as measured by hematology and blood biochemistry parameters (Supporting Information S1: Tables S3 and S4). A second tolerability study including only the two highest doses (0.18 and 0.24 mg/kg) confirmed that PVEK was well tolerated with no severe weight loss or acute toxicity observed (Supporting Information S1: Figure S1B and Supporting Information S1: Tables S5 and S6).

**Figure 2:**
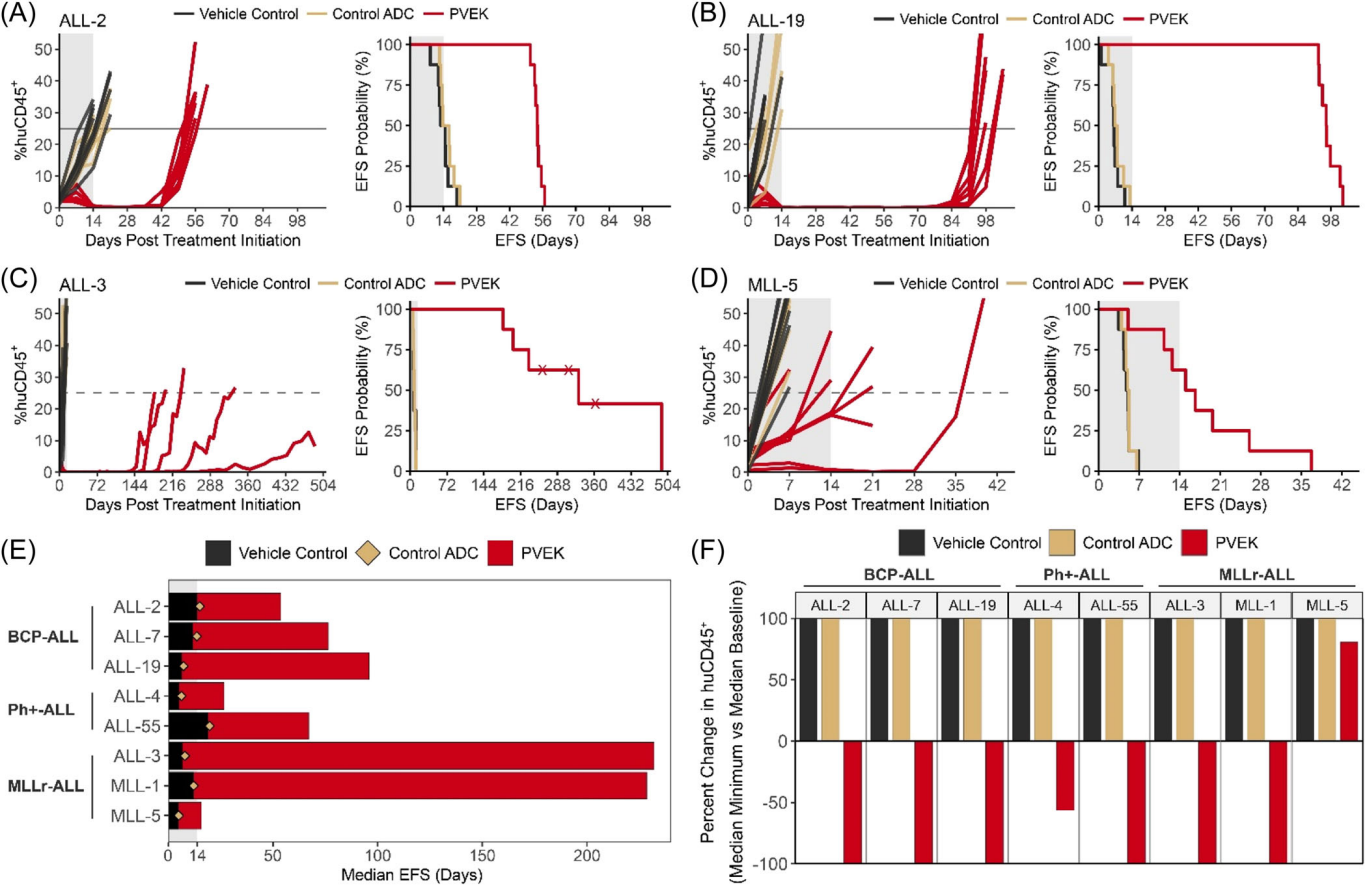
**In vivo responses of pediatric ALL PDXs to PVEK in the conventional study**. Leukemia burden was measured by enumeration of human CD45^+^ (%huCD45^+^) cells in the PB over time. PVEK treatment commenced once the %huCD45^+^ was ≥1% **(A–D)**. Representative engraftment (left) and survival (right) plots of ALL‐2 **(A)**, ALL‐19 **(B)**, ALL‐3 **(C)**, and MLL‐5 **(D)**. **(E)** Swimmer plot of median EFS of eight ALL PDXs in response to vehicle control, control ADC, or PVEK (0.24 mg/kg) treatment. **(F)** Waterfall plot displaying the percent change in huCD45^+^ cells from baseline, capped at +100%. ADC, antibody–drug conjugate; black lines/bars, vehicle‐treated; dashed line, event threshold; EFS, event‐free survival; gold lines/diamond/bars, control ADC‐treated; red lines/bars, PVEK‐treated; shaded area, treatment period; X, censored data.

Initial in vivo efficacy testing of PVEK was carried out against eight PDXs consisting of three BCP‐ALL (ALL‐2, ALL‐7, ALL‐19), three MLLr‐ALL (ALL‐3, MLL‐1, MLL‐5), and two Ph^+^‐ALL (ALL‐4, ALL‐55) PDXs (Figure 2A–D, Supporting Information S1: Figure S2 and Table 1). Median EFS in the PVEK‐treated groups ranged from 15.8 (MLL‐5) to 232 (ALL‐3) days (T‐C 10.9–225 days; T/C 3.2–34.6), with mice engrafted with seven out of eight PDXs achieving a median EFS well beyond the 3‐week treatment period and two PDXs (ALL‐3, MLL‐1) beyond 200 days (Figure 2E). All eight PDXs tested had significantly delayed disease progression following PVEK treatment compared with vehicle control‐treated groups, with six PDXs achieving maximum regression (100% reduction in %huCD45^+^ cells in the PB) at a minimum of one timepoint following the initiation of PVEK treatment (Figure 2F).

**Table 1:** Summary of the in vivo results from the conventional efficacy study with PVEK.

Subtype	PDX	Treatment group	EFS (days)	EFS T‐C (days)	EFS T/C (days)	*p*‐value vs. vehicle control	ORM
BCP‐ALL	ALL‐2	Vehicle control	13.8				
		Control ADC	15.1	1.3	1.1	0.2065	PD1
		PVEK	53.7	39.9	3.9	0.0002	MCR
	ALL‐7	Vehicle control	11.7				
		Control ADC	13.7	2.0	1.2	0.0069	PD1
		PVEK	76.4	64.7	6.5	0.0002	MCR
	ALL‐19	Vehicle control	6.3				
		Control ADC	7.3	1.0	1.2	0.5646	PD1
		PVEK	96.0	89.7	15.2	0.0002	MCR
Ph^+^‐ALL	ALL‐4	Vehicle control	5.0				
		Control ADC	6.3	1.3	1.3	0.0115	PD1
		PVEK	26.7	21.7	5.3	0.0005	PD2
	ALL‐55	Vehicle control	19.0				
		Control ADC	19.7	0.7	1.0	0.8754	PD1
		PVEK	67.2	48.2	3.5	0.0002	MCR
MLLr‐ALL	ALL‐3	Vehicle control	6.7				
		Control ADC	8.1	1.4	1.2	0.7538	PD1
		PVEK	232	225	34.6	0.0029	MCR
	MLL‐1	Vehicle control	12.2				
		Control ADC	12.2	0	1.0	0.9173	PD1
		PVEK	229	217	18.8	0.0011	MCR
	MLL‐5	Vehicle control	4.9				
		Control ADC	5.1	0.2	1.0	0.4592	PD1
		PVEK	15.8	10.9	3.2	0.0012	PD2

*Note*: Includes vehicle control, control ADC, and PVEK‐treated groups for eight ALL PDXs.

Abbreviations: EFS, event‐free survival; ORM, objective response measure; T‐C, treatment minus control; T/C, treatment divided by control.

In terms of stringent objective response criteria modeled after the clinical setting, six out of eight PDXs scored the highest ORM of maintained complete response (MCR), including all three BCP‐ALLs (ALL‐2, ALL‐7, ALL‐19), two MLLr‐ALLs (ALL‐3 and MLL‐1), and one Ph^+^‐ALL (ALL‐55) with the other two PDXs (one MLLr‐ALL, MLL‐5, and one Ph^+^‐ALL, ALL‐4) scoring progressive disease 2 (PD2; Table 1). Of note, two of the BCP‐ALL PDXs harbor structural variants in *TCF3::HLF* (ALL‐7) or *NUP214::ABL1* (ALL‐19), which are associated with a higher risk of poor outcome (Supporting Information S1: Table S1), while ALL‐2 was derived from a patient who underwent multiple relapses and died from their disease.^23^, ^24^ Moreover, three of five MCRs were elicited in PDXs derived from high‐risk pediatric ALL subtypes (one Ph^+^‐ALL and two MLLr‐ALL PDXs). No mice were lost due to treatment‐related toxicity and, importantly, the control ADC exerted negligible effects on the progression of all PDXs compared with vehicle control‐treated mice (Figure 2, Supporting Information S1: Figure S2 and Table 1). Of note, the PDX with the highest CD123 mRNA and cell surface expression (MLL‐5) exhibited the poorest response to PVEK. Complete summary data for the efficacy experiment are described in Supporting Information S1: Table S7.

**Figure 3:**
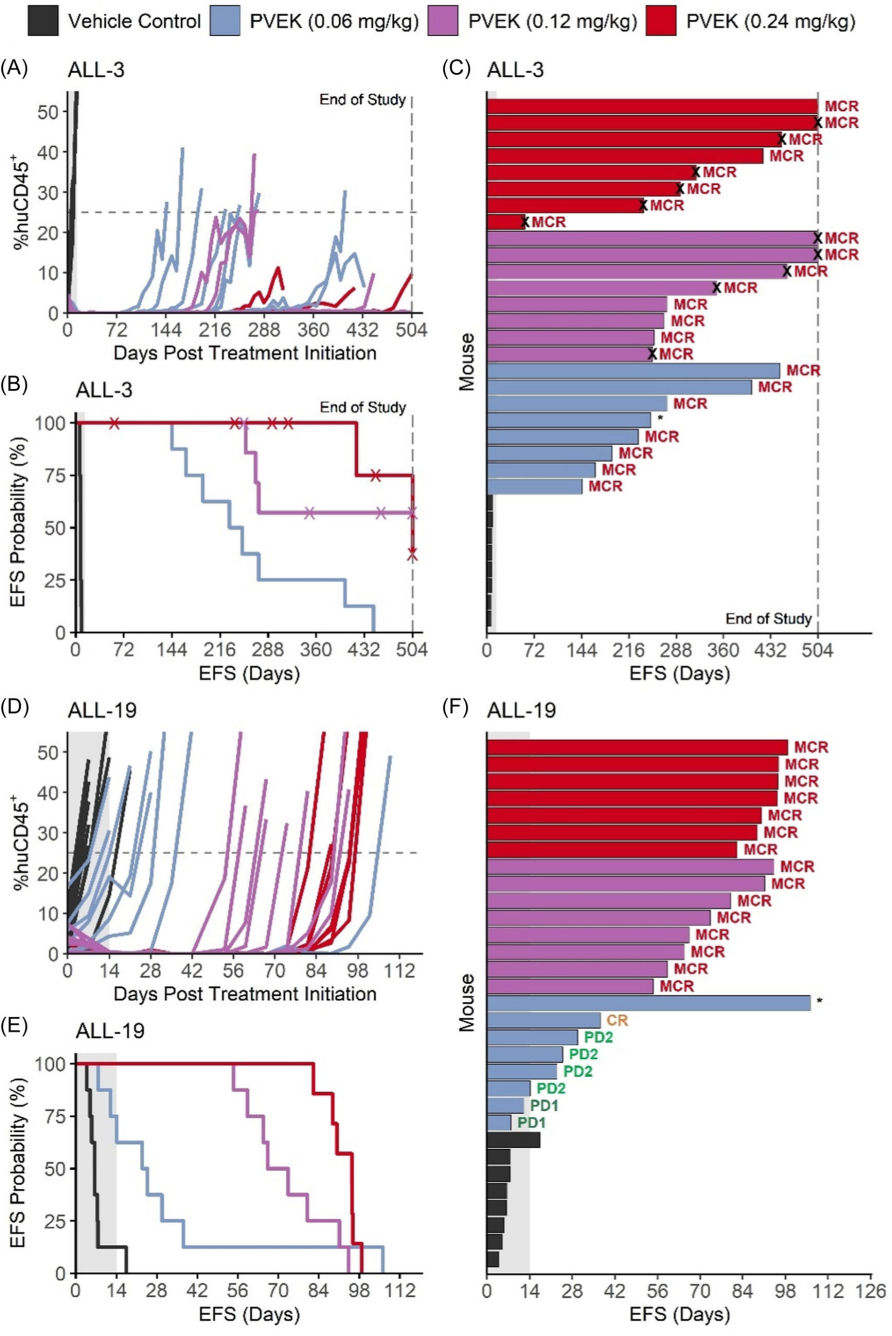
**In vivo efficacy of PVEK in the dose escalation study**. ALL‐3 and ALL‐19 were treated with 0.06 (blue), 0.12 (purple), or 0.24 (red) mg/kg PVEK, or with a vehicle control (black) IV weekly × 3 weeks. Leukemia burden was measured by enumeration of human CD45^+^ (%huCD45^+^) cells in the PB. Engraftment **(A**, **D)**, survival **(B**, **E)**, and swimmer **(C**, **F)** plots for ALL‐3 and ALL‐19, respectively. Each line/bar on engraftment **(A**, **D)** and swimmer plots **(C**, **F)** represents one mouse. CR, complete remission; EFS, event‐free survival; horizontal dashed line, event threshold; shaded area, treatment period; MCR, maintained complete response; PD1, progressive disease 1; PD2, progressive disease 2; vertical dashed line, end of study; X, censored data; *, objective response measure not determined due to low %huCD45^+^ at treatment initiation.

Since PVEK exerted impressive single‐agent in vivo efficacy at 0.24 mg/kg against aggressive orthotopic PDX models of pediatric ALL, it was important to evaluate its activity across a broad dose range. Therefore, a dose escalation study was undertaken consisting of three PVEK doses as low as 25% of the company‐recommended dose (0.06, 0.12, and 0.24 mg/kg). ALL‐3 and ALL‐19 were selected for this study as they represent high‐risk pediatric ALL subtypes. At all three doses, PVEK significantly prolonged the EFS (T‐C) of mice engrafted with both PDXs compared with vehicle control‐treated mice by a median of between 17.4 and 311 days (T/C 3.5–65.1) (Figure 3 and Supporting Information S1: Table S8). For ALL‐3, PVEK prolonged mouse EFS by a median of 233–311 days (T/C 34.8–65.1) with each dose eliciting MCRs (Figure 3A–C, Supporting Information S1: Table S8). For ALL‐19, PVEK prolonged mouse EFS by a median of 17.4–88.9 days (T/C 3.5–14.7) with treatment at 0.12 and 0.24 mg/kg achieving an MCR in all mice, while the 0.06 mg/kg dose elicited a median objective response of PD2 (Figure 3D–F and Supporting Information S1: Table S8). Complete data for the dose escalation study are described in Supporting Information S1: Table S9. The responses observed at the 0.24 mg/kg dose were strikingly consistent between the conventional and dose escalation studies, despite them being performed more than a year apart (Supporting Information S1: Figure S3 and Supporting Information S1: Tables S8 and S9). These findings demonstrate that PVEK exerts in vivo activity against pediatric ALL PDXs over at least a fourfold dose range.

**Figure 4:**
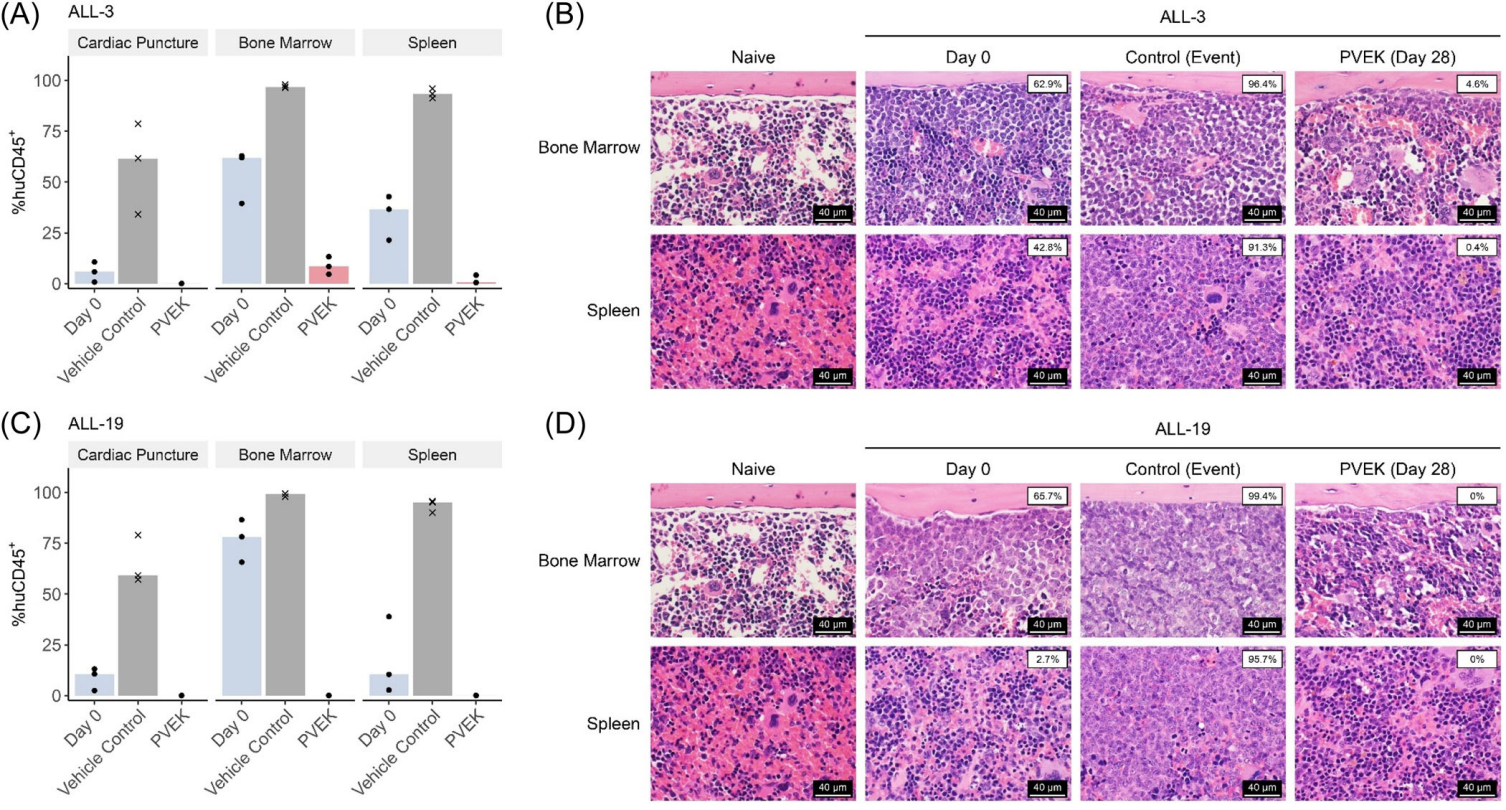
**Leukemic infiltration in hematolymphoid organs following pivekimab sunirine (PVEK) treatment**. Infiltration of leukemia cells was measured by the enumeration of human CD45^+^ (%huCD45^+^) cells in cardiac puncture, bone marrow (BM), and spleen samples at event or Day 28 (whichever occurred first). Infiltration plots of ALL‐3 **(A)** and ALL‐19 **(C)**. Black dots represent samples from individual mice culled at Day 28. Black crosses represent mice that reached event before Day 28. Bars represent median values within each group. Representative H&E stained sections of the spleen and femoral bone marrow of ALL‐3 **(B)** and ALL‐19 **(D)** demonstrating a reduction in tumor burden following administration of PVEK. For comparison, the images of naïve NSG mouse spleen and BM are reproduced in both **(B)** and **(D)**. White boxes indicate the %huCD45^+^ cells measured by flow cytometry. Scale bar, 40 µm.

ALL is primarily a disease of the BM, therefore we next assessed the effects of PVEK on leukemia infiltration of the femoral BM and spleen at a discreet timepoint following treatment initiation (Day 28 or event, whichever occurred first). PVEK treatment resulted in near complete eradication of huCD45^+^ cells in the blood, spleen, and BM in two B‐lineage ALL PDXs (ALL‐3 and ALL‐19) compared with vehicle control‐treated mice as determined by both flow cytometry and histopathological examination (Figure 4).

**Figure 5:**
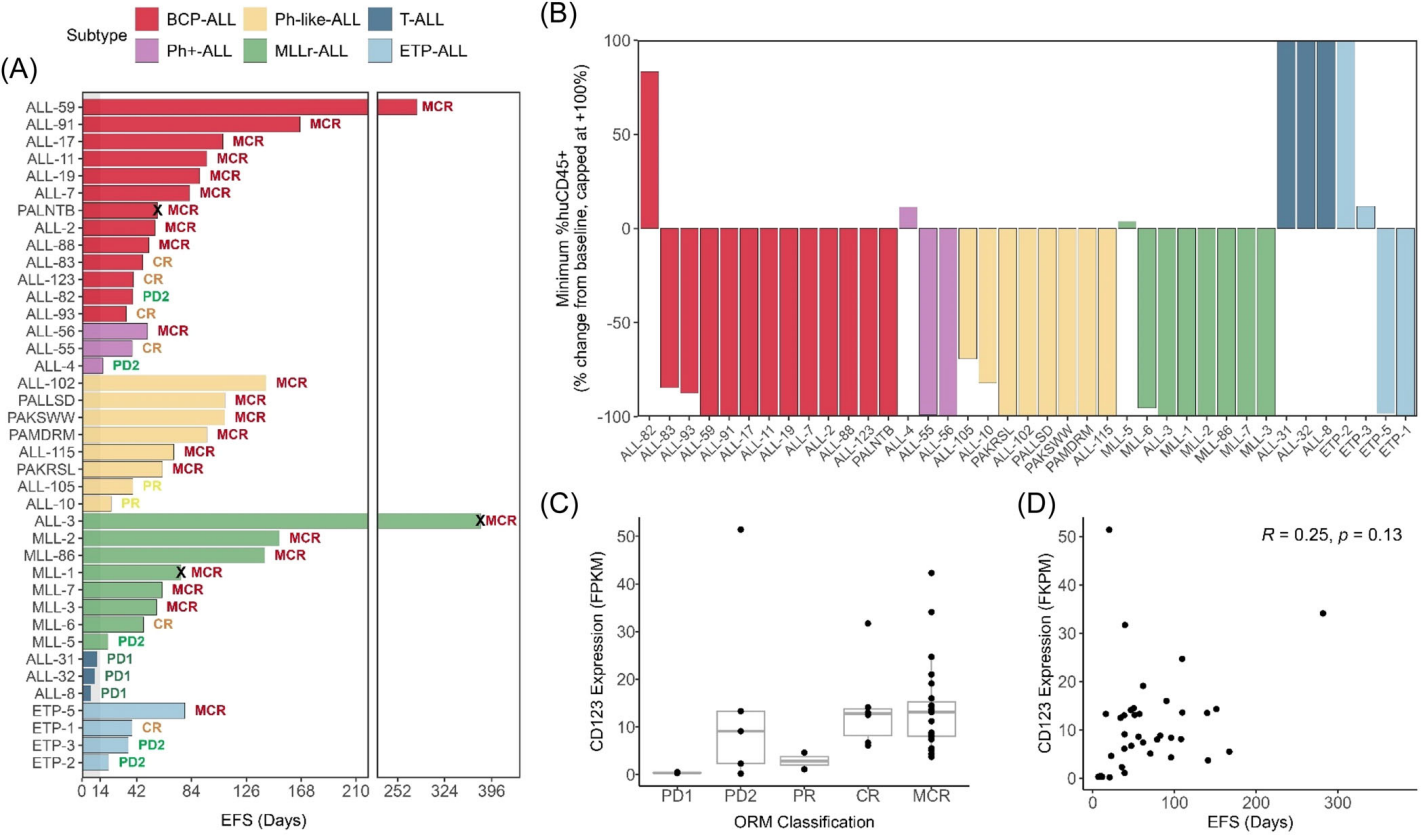
**In vivo responses of pediatric ALL PDXs to PVEK in the SMT**. Leukemia burden was measured by enumeration of human CD45^+^ (%huCD45^+^) cells in the PB. PVEK treatment commenced once when mice reached ≥1% %huCD45^+^. **(A)** Swimmer plots of 39 PDXs colored by subtype and ordered by longest to shortest EFS within each subtype. **(B)** Waterfall plot displaying the percent change in huCD45^+^ cells from baseline in 39 PDXs, capped at +100%. Colored by subtype and ordered by worst to best responders within each subtype. **(C)** CD123 messenger RNA (mRNA) expression of 39 PDXs separated by objective response measure scores. **(D)** Correlation between EFS and CD123 mRNA expression. CR, complete remission; MCR, maintained complete response; *p*, *p*‐value; PD1, progressive disease 1; PD2, progressive disease 2; PDX, patient‐derived xenograft; PR, partial response; *R*, Pearson's correlation coefficient; shaded area, treatment period; X, censored data.

To further investigate the breadth of in vivo responses to PVEK across a range of ALL subtypes, PVEK was evaluated against a panel of 40 PDXs (33 B‐cell lineage consisting of BCP‐ALL, Ph^+^‐ALL, Ph‐like ALL, and MLLr‐ALL; 7 T‐cell lineage consisting of T‐ALL and ETP‐ALL) in SMT format. PDXs were selected based on CD123 mRNA expression (Figure 1A). Following the initiation of PVEK treatment, mouse EFS ranged from 6.8 (ALL‐8) to 379 (ALL‐3) days with a median EFS of 57.2 days across all PDXs tested (Figure 5A and Table 2). Thirty‐one PDXs scored objective responses (12 out of 13 BCP‐ALL, two out of three Ph^+^‐ALL, eight out of eight Ph‐like‐ALL, seven out of eight MLLr‐ALL, zero out of three T‐ALL, two out of four ETP‐ALL), with a total of 23 MCRs, six CRs, and two PRs (Figure 5A,C, Table 2, Supporting Information S1: Figures S4 and S5A). Regression of leukemia (huCD45^+^) cells in the PB after initiation of PVEK treatment was observed in 31/39 PDXs, with 23 showing maximum regression (−100%) of leukemic burden in the PB (Figure 5B). The SMT also revealed that PVEK was highly effective against many BCP‐ALL PDXs that harbor molecular aberrations associated with higher risk.^23^, ^24^, ^25^, ^26^ These included multiple *IKZF1*‐ (ALL‐17, ALL‐19, ALL‐88, ALL‐93) or PAX5‐ (ALL‐11, ALL‐88) altered PDXs, one *TCF3::HLF* fusion (ALL‐7), and one *NUP214::ABL1* fusion (ALL‐19).

**Table 2:** Summary of the in vivo efficacy of PVEK in a single‐mouse trial against ALL PDXs.

Subtype	PDX	CD123 mRNA expression (FPKM)	EFS (days)	AOC	ORM
BCP‐ALL	ALL‐59	34.1	282	258	MCR
	ALL‐91	5.5	167	156	MCR
	ALL‐17	8.1	108	101	MCR
	ALL‐11	4.3	95.8	89.1	MCR
	ALL‐19	16.0	90.2	84.5	MCR
	ALL‐7	8.8	82.5	74.6	MCR
	PALNTB	42.3	58	*	MCR
	ALL‐2	8.6	56.0	48.2	MCR
	ALL‐88	13.1	51.3	45.6	MCR
	ALL‐83	14.1	46.6	38.7	CR
	ALL‐123	31.7	39.4	33.9	CR
	ALL‐82	9.1	38.9	30.3	PD2
	ALL‐93	12.6	28.0	31.9	CR
Ph^+^‐ALL	ALL‐56	14.5	50.2	45.1	MCR
	ALL‐55	6.1	38.6	32.2	CR
	ALL‐4	13.3	16.0	6.3	PD2
Ph‐like ALL	ALL‐102	3.7	141	121	MCR
	PALLSD	13.6	110	101	MCR
	PAKSWW	24.7	109	100	MCR
	PAMDRM	8.4	95.9	87.5	MCR
	ALL‐115	5.1	70.4	63.4	MCR
	PAKRSL	19.1	61.6	52.5	MCR
	ALL‐105	1.1	39.0	32.1	PR
	ALL‐10	4.6	22.5	16.3	PR
MLLr‐ALL	ALL‐3	11.8	379	*	MCR
	MLL‐2	14.3	151	144	MCR
	MLL‐86	13.5	140	132	MCR
	MLL‐1	21.0	76	*	MCR
	MLL‐7	7.4	61.5	56.5	MCR
	MLL‐3	13.3	57.2	52.2	MCR
	MLL‐6	6.7	46.4	40.5	CR
	MLL‐5	51.4	20.1	12.2	PD2
T‐ALL	ALL‐31	0.3	11.5	5.0	PD1
	ALL‐32	0.5	9.8	4.8	PD1
	ALL‐8	0.3	6.8	1.7	PD1
ETP‐ALL	ETP‐5	8.0	78.8	63.4	MCR
	ETP‐1	13.0	38.4	33.8	CR
	ETP‐3	2.3	35.6	26.4	PD2
	ETP‐2	0.2	20.6	14.5	PD2

*Note*: A panel of 39 PDXs was utilized, where each PDX was inoculated into a single mouse.

Abbreviations: AOC, area over the curve; EFS, event‐free survival; FPKM, fragments per kilobase of transcript per million mapped reads; ORM, objective response measure; *, AOC not determined due to non‐leukemia related event.

Despite the specificity of PVEK for CD123, we observed only a moderate correlation between mouse EFS and CD123 RFI (Pearson's *R* = 0.49, *p* = 0.0086) and no correlation between ORMs/EFS and CD123 mRNA (Figure 5C,D and Supporting Information S1: Figure S5). However, consistent with the significantly higher CD123 expression for B‐lineage compared with T‐lineage ALL PDXs reported above, mouse EFS was significantly longer for B‐lineage PDXs (*n* = 31) compared with T‐lineage PDXs (*n* = 7; *p* < 0.0001). In the above analysis, the MLL‐8 mouse was euthanized due to developing a mouse lymphoma during the treatment window and was therefore excluded from this study. The mouse engrafted with the PALNTB PDX was euthanized at Day 58 due to an undefined non‐leukemia‐related illness, the MLL‐1 mouse was euthanized at Day 62 due to a mouse‐related thymoma, while the ALL‐3 mouse (the best responder of all PDXs) was euthanized at Day 379 due to a prolapsed bowel. Therefore, these mice were excluded from analysis in Figure 5D and Supporting Information S1: Figure S5B. A comparison of data obtained across the conventional, dose escalation, and SMT studies where the same PDXs were evaluated demonstrated a high degree of concordance, despite the studies having been carried out over more than a 2‐year span (Supporting Information S1: Figure S6 and Supporting Information S1: Table S10).

### Ex vivo cytotoxicity of the PVEK payload DGN549

The above studies highlighted that CD123 expression is required, but not sufficient, for the in vivo activity of PVEK against pediatric ALL PDXs. Despite MLL‐5 having the highest CD123 mRNA and cell surface expression of all 90 PDXs included in this study, it had one of the poorest responses to PVEK in vivo. Therefore, ex vivo cytotoxicity assays were carried out on the eight PDXs used in the original conventional efficacy study (Figure 2 and Supporting Information S1: Figure S2) to determine whether MLL‐5 exhibited high‐level resistance to the PVEK payload, DGN549. IC_50_ values for the eight PDXs ranged from 0.93 to 91.2 nM (Supporting Information S1: Figure S7 and Supporting Information S1: Table S11), suggesting that all PDXs were highly sensitive to the payload. MLL‐5 (IC_50_ = 3.7 nM) had the second lowest IC_50_, behind only ALL‐7 (IC_50_ = 0.93 nM). Given that MLL‐5 was one of the more sensitive PDXs to DGN549 ex vivo, we hypothesized that the basis for the poor in vivo response of MLL‐5 to PVEK could be due to lack of receptor internalization upon ligation with the G4723A antibody.

### Internalization of G4723A by pediatric ALL PDX cells ex vivo

**Figure 6:**
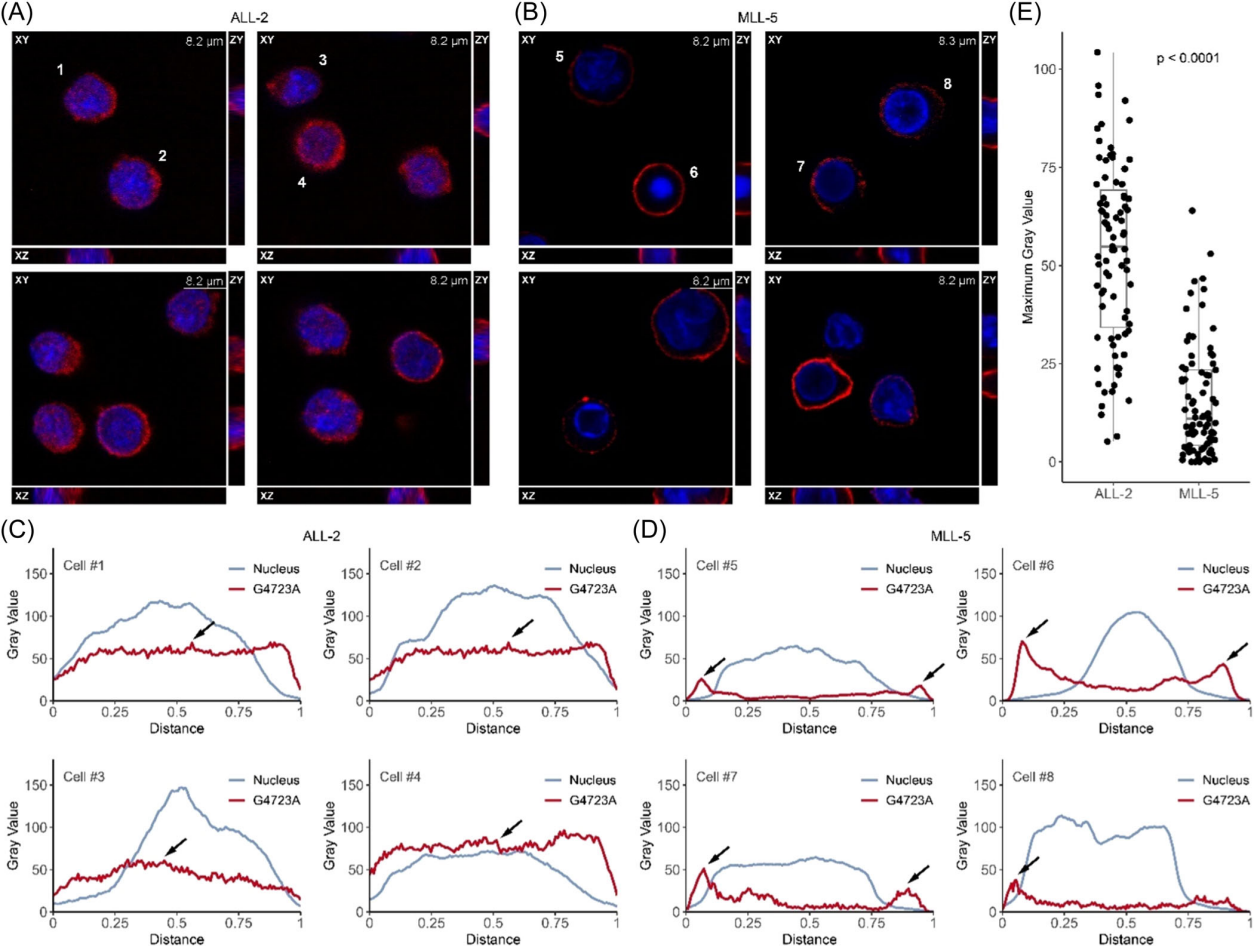
**Internalization of G4723A by ALL‐2 and MLL‐5 cells ex vivo**. Internalization of the G4723A antibody was evaluated by confocal microscopy. Representative orthogonally viewed images of ALL‐2 **(A)** and MLL‐5 **(B)**. Red color indicates the AF™ 647 fluorescence, corresponding to the G4723A antibody; blue color indicates Hoechst 33342 fluorescence, corresponding to the nuclei; numbers represent cells plotted in **(C**, **D)**. Plot profiles of AF™ 647 and Hoechst 33342 representing G4723A and nuclei, respectively, in ALL‐2 **(C)** and MLL‐5 **(D)** cells. In ALL‐2 cells, arrows indicate a high G4723A signal that overlays the nuclei signal, suggesting internalized G4723A antibody. In MLL‐5 cells, arrows show an accumulation of G4723A signal outside the nuclei, suggesting a lack of internalization of the G4723A antibody. Red line, G4723A; blue line, nuclei. **(E)** Comparison of the maximum overlaying gray values of AF™ 647 and Hoechst 33342 fluorescence in ALL‐2 and MLL‐5 cells. The *p*‐value (*p* < 0.0001) was calculated with the Wilcoxon Rank‐Sum Test.

To test whether lack of receptor internalization could explain the poor in vivo response of MLL‐5 to PVEK, we compared internalization of the G4723A antibody in two PDXs with high CD123 expression but diverse in vivo responses to PVEK (ALL‐2, T‐C 40 days, MCR; MLL‐5, T‐C 11 days, PD2; Figures 1 and 2; Table 1). Confocal microscopy analysis and *Z*‐stacking revealed that ALL‐2 cells efficiently internalized the G4723A antibody with a median maximum overlaying gray value of 54.9 (5.2–104.3; Figure 6A,C,E). In contrast, the G4723A antibody remained distinctly around the periphery of almost all MLL‐5 cells, with a median maximum overlaying gray value of 11.0 (0–64.0; Figure 6B,D,E), which was significantly lower than ALL‐2 (*p* < 0.0001). These findings indicate that the G4723A antibody may be trapped on the surface of MLL‐5 cells, thereby preventing the DGN549 payload from reaching its intracellular target(s).

## DISCUSSION

The high cure rates that have been achieved in pediatric ALL over the last few decades have shifted the clinical emphasis toward how to manage high‐risk and relapsed/refractory disease. Reflecting this change in focus, the panel of pediatric ALL PDXs used in this study accentuates high‐risk patients either at diagnosis or relapse, in which standard‐of‐care chemotherapy was not curative.^15^ The impressive single‐agent in vivo activity of PVEK against a broad range of B‐lineage ALL PDXs observed in this study, across a fourfold dose range, suggests that PVEK may prove to be of clinical utility in the high‐risk or relapsed/refractory setting in pediatric ALL. These results are in contrast to the majority of single agents tested by the NCI‐funded pediatric preclinical testing consortia (PPTP, PPTC, PIVOT),^27^ further highlighting the potential promise of PVEK.

Following earlier studies reporting strong responses to PVEK in BPDCN and AML PDX models in vivo, ongoing phase I/II clinical trials investigating both diseases (NCT03386513, NCT04086264) have reported similarly positive results.^9^, ^10^, ^11^, ^28^, ^29^ These findings support the relevance of utilizing preclinical models of leukemia expressing CD123 as a tool for clinical decision‐making.

As anticipated, PVEK was ineffective against ALL PDXs with minimal CD123 expression. This was particularly evident in the T‐lineage ALL PDXs, where objective responses were only observed in two ETP‐ALLs, both with considerably higher CD123 mRNA expression than other T‐lineage PDXs. The significantly higher CD123 expression in B‐lineage compared with T‐lineage ALL PDXs translated to significantly better in vivo PVEK responses when these two groups were compared. We observed a moderate correlation between CD123 cell surface expression and in vivo PVEK responses within the group of B‐lineage PDXs, but no significant correlation with basal mRNA expression, highlighting the potential for using cell surface expression as a predictor of response in the clinic.

Our analysis suggested that CD123 is highly expressed in the hyperdiploid subtype compared to other BCP‐ALLs, in agreement with earlier studies.^5^, ^6^ Hyperdiploid is the most common genetic subtype in pediatric B‐ALL, occurring in 25%–35% of patients.^30^ However, this proportion was not reflected in our PDX panel, which focused on high‐risk and/or poor‐outcome cases. Despite a generally favorable prognosis, relapse is common in this subtype and represents up to 29% of relapsed B‐ALL cases.^31^ Although our panel did not include any matched diagnosis and relapsed PDXs from the same hyperdiploid patient, one PDX predicted to be hyperdiploid was derived from a patient after third relapse (ALL‐2) and still exhibited an excellent response to PVEK (MCR). Furthermore, in our study, the longest remission achieved among the BCP‐ALL PDXs was in a hyperdiploid PDX (ALL‐59), which taken together suggests this subtype of ALL may be particularly responsive to PVEK.

Several PDXs with high CD123 expression exhibited resistance to PVEK in vivo, the most striking of which was MLL‐5 with the highest CD123 mRNA and cell surface expression of all 90 PDXs. MLL‐5 was acutely sensitive ex vivo to the PVEK payload, DGN549, and evidence suggests a defect in receptor internalization as the basis for MLL‐5 resistance to PVEK. Future studies will focus on defining the underlying mechanism of defective CD123 internalization in MLL‐5. Furthermore, antigen loss has been observed following CAR T‐cell therapy targeting CD123 in BPDCN, in which CD123‐negative populations were identified either lacking CD123 DNA or expressing isoforms without the required epitope, and may be an alternative mechanism which can lead to PVEK resistance.^32^ Overall, the findings of our study with a large cohort of pediatric ALL PDXs indicate that CD123 expression is necessary, but not sufficient, for the in vivo efficacy of PVEK.

In the SMT experiment, for one pair of BCP‐ALL PDXs derived from the same patient at diagnosis and relapse (ALL‐82 and ALL‐83, respectively), an objective response was only observed in the PDX derived at relapse whereas that at diagnosis achieved a PD2. Furthermore, matched PDXs established from a patient with Ph‐like ALL at diagnosis (ALL‐102), first relapse (ALL‐105), and third relapse (ALL‐115) achieved objective responses of MCR in the diagnosis and third relapse samples but only a PR in the first relapse PDX (ALL‐105). In both sets of PDXs, when comparing PDXs established from the same patient, the poorest response was observed where CD123 expression was the lowest. It was previously reported that CD123 expression is often increased in relapsed BCP‐ALL samples compared to diagnosis, which we observed between ALL‐82 and ALL‐83.^6^ Therefore, at least in BCP‐ALL, PVEK may prove to be an effective second‐line treatment option.

In recent years, several options have emerged for treating high‐risk ALL subtypes including targeted agents such as tyrosine kinase inhibitors (imatinib, dasatinib) for Ph^+^‐ALL and menin inhibitors (e.g., revumenib) for MLLr‐ALL, as well as CD19‐targeting CAR‐T cell therapy and the bispecific T‐cell engager (BiTE) blinatumomab for relapsed/refractory patients.^33^, ^34^, ^35^, ^36^, ^37^, ^38^ Unfortunately, the outcome for these patients still falls short of what has been achieved in standard risk ALL and there remains a need for additional therapies that can improve upon the options currently available. Targeting CD123 appears to be a promising strategy in acute leukemias, as a CD3‐CD123 BiTE has also been developed for treating AML and may prove to be useful against high‐risk ALL.^39^ There is potential for PVEK to be complementary to a CD123‐targeting BiTE as it does not rely on the activity of normal T‐cells and using them in combination may further enhance their potency. The exceptional single‐agent responses to PVEK observed in this study across multiple PDXs derived from high‐risk pediatric B‐ALL subtypes, including Ph^+^‐ALL, Ph‐like‐ALL, and MLLr‐ALL, suggest that PVEK warrants further clinical evaluation to determine whether it can improve the outcome of high‐risk pediatric B‐ALL.

## AUTHOR CONTRIBUTIONS

Ben Watts involved in data curation, formal analysis, investigation, visualization, methodology, writing—original draft, writing—review and editing. Christopher M. Smith involved in data curation, software, formal analysis, visualization, methodology, writing—original draft, writing—review and editing. Kathryn Evans involved in conceptualization, data curation, formal analysis, validation, investigation, methodology, writing—review and editing. Andrew Gifford involved in formal analysis, methodology, writing—review and editing. Sara M.A. Mohamed involved in data curation, formal analysis, visualization, methodology, writing—review and editing. Stephen W. Erickson involved in formal analysis, visualization, writing—review and editing. Eric J. Earley involved in formal analysis, visualization, writing—review and editing. Steven Neuhauser involved in formal analysis, visualization, writing—review and editing. Timothy M. Stearns involved in formal analysis, visualization, writing—review and editing. Vivek M. Philip involved in formal analysis, visualization, writing—review and editing. Jeffrey H. Chuang involved in resources, project administration, funding acquisition. Patrick A. Zweidler‐McKay involved in conceptualization, resources, writing—review and editing. Sribalaji Lakshmikanthan involved in conceptualization, resources, writing—review and editing. Emily L. Jocoy involved in project administration, writing—review and editing. Carol J. Bult involved in resources, project administration, writing—review and editing, funding acquisition. Beverly A. Teicher involved in resources, project administration, writing—review and editing. Malcolm A. Smith involved in conceptualization, resources, project administration, writing—review and editing, funding acquisition. Richard B. Lock involved in conceptualization, resources, writing—original draft, project administration, writing—review and editing, supervision, funding acquisition.

## CONFLICT OF INTEREST STATEMENT

Patrick A. Zweidler‐McKay and Sribalaji Lakshmikanthan report employment at ImmunoGen, Inc. at the time of analysis and reporting. All other authors declare no conflicts of interest.

## ETHICS STATEMENT

All animal experiments received prior approval from the Animal Care and Ethics Committee of UNSW Sydney (Sydney, NSW, Australia).

## FUNDING

This research was funded by grants from the National Cancer Institute (CA199000, CA199222, and CA263963), a fellowship from the National Health and Medical Research Council of Australia (APP1157871) to RBL, and the Annie Frida Minna Adams Charitable Trust, the Hillcrest Foundation, and the Alma Hazel Eddy Trust. Children's Cancer Institute Australia is affiliated with UNSW Sydney and The Sydney Children's Hospitals Network.

## Supporting information

Supporting information.

## Data Availability

The data generated in this study are available upon request from the corresponding author.
