# Supplementary material for: The CD123 antibody–drug conjugate pivekimab sunirine exerts profound activity in preclinical models of pediatric acute lymphoblastic leukemia
Source: Hemasphere. 2025 Jan 17;9(1):e70063. doi: 10.1002/hem3.70063 (PMC11739898; doi:10.1002/hem3.70063)
Supplement: Supplementary file 1 — Supporting information. [file HEM3-9-e70063-s001.docx]

**THE CD123 ANTIBODY-DRUG CONJUGATE PIVEKIMAB SUNIRINE EXERTS PROFOUND ACTIVITY IN PRECLINICAL MODELS OF PEDIATRIC ACUTE LYMPHOBLASTIC LEUKEMIA**

Ben Watts^1^*, Christopher M. Smith^1^*, Kathryn Evans^1^, Andrew J. Gifford^1,2,3^, Sara M.A. Mohamed^1,4^, Stephen W. Erickson^5^, Eric J. Earley^5^, Steven Neuhauser^6^, Timothy M. Stearns^6^, Vivek M. Philip^6^, Jeffrey H. Chuang^7^, Patrick A. Zweidler-McKay^8^, Sribalaji Lakshmikanthan^8^, Emily L. Jocoy^9^, Carol J. Bult^6^, Beverly A. Teicher^10^, Malcolm A. Smith^10^ and Richard B. Lock^1^

*These authors contributed equally to this work

^1^Children's Cancer Institute, Lowy Cancer Research Centre, School of Clinical Medicine, UNSW Medicine & Health, UNSW Centre for Childhood Cancer Research, UNSW Sydney, Sydney, NSW, Australia

^2^Anatomical Pathology, NSW Health Pathology, Prince of Wales Hospital, Randwick, NSW, Australia

^3^School of Clinical Medicine, UNSW Medicine & Health, UNSW Sydney, Sydney, NSW, Australia

^4^Department of Pharmaceutics and Industrial Pharmacy, Faculty of Pharmacy, Ain Shams University, Cairo, Egypt

^5^RTI International, Research Triangle Park, NC

^6^The Jackson Laboratory for Mammalian Genetics, Bar Harbor, ME

^7^The Jackson Laboratory for Genomic Medicine, Farmington, CT

^8^ImmunoGen, Inc., Waltham, MA

^9^The Jackson Laboratory, Sacramento, CA

^10^National Cancer Institute, Bethesda, MD

**SUPPLEMENTARY INFORMATION**

**SUPPLEMENTARY METHODS**

**Statistical Methods for Evaluating Treatment Response**

The exact time-to-event is estimated by interpolating between the measurements directly preceding and following the event, assuming log-linear growth. Differences in event-free survival (EFS) between experimental groups (e.g., treated vs controls) are tested using the *G^ρ^* test of Harrington & Fleming (*Biometrika* 69:553-566, 1982; *α* = 0.05, two-sided alternative) with *ρ* = 1, which is equivalent to the Peto & Peto modification of Gehan-Wilcoxon.

The *objective response measure* (ORM) categories are progressive disease (PD, which is subdivided into progressive disease without and with growth delay, PD1 and PD2 respectively, defined only for treated mice), stable disease (SD), partial response (PR), complete response (CR), and maintained complete response (MCR).

- PD when %huCD45 never < 1% during study period and mouse reaches event (%huCD45 > 25%) at some point during the study period,
- PD1 when PD, and the mouse’s time to event ≤ 200% the median time-to-event in control group,
- PD2 when PD, but the mouse’s time-to-event > 200% the median time-to-event in control group,
- SD when %huCD45 never < 1% and mouse never reaches event during the study period (42 days),
- PR when %huCD45 < 1% at least once during the study period, but not CR,
- CR when %huCD45 < 1% for at least 2 consecutive weekly readings during the study period, regardless of whether event is reached at a later time-point, and
- MCR when %huCD45 < 1% for at least 3 consecutive weekly readings at any time after treatment has been completed.

Overall group response is determined by the median response among evaluable mice as follows: Each individual mouse is assigned a score from 0 to 10 based on their response: PD1=0, PD2=2, SD=4, PR=6, CR=8, and MCR=10, and the median for the group determines the overall response. If the median score is half-way between an objective response number category, the objective response is assigned to the lower response category (e.g., an objective response score of 9 is scored CR). Studies in which toxicity is greater than 25%, or in which the control group is not SD or worse, are considered inevaluable and are excluded from analysis. Treatment groups with PR, CR, or MCR are considered to have had an objective response. Agents inducing objective responses are considered highly active against the tested line, while agents inducing SD or PD2 are considered to have intermediate activity, and agents producing PD1 are considered to have a low level of activity against the tested line.

Summary tables for efficacy experiments will summarize results for each experimental group (e.g., treated vs controls) and include the following columns:

- PDX, the alphanumeric code for the tumor model,
- Grp, experimental group, usually C (Control) or T (Treatment)
- *N*, the total number of mice entering experiment,
- *N_d_*, the number of mice experiencing toxic death,
- *N_x_*, the number of additional mice excluded from analysis,
- *N_a_*, the number of mice in analysis,
- *N_ev_*, the number of events,
- KMmed, the Kaplan-Meier estimate of median time-to-event (days),
- EFS T-C, the difference in median time-to-event (days) between T and C groups,
- EFS T/C, the ratio of median time-to-event between T and C groups,
- EFS *p*-value, computed using the Gehan-Wilcoxon test,
- Baseline %huCD45^+^ mean+SD, the mean + standard deviation of the per-mouse baseline %huCD45^+^,
- %huCD45^+^ *p*-value between groups A and B at baseline, computed using Wilcoxon rank sum test
- min %huCD45^+^ mean±SD, the mean ± standard deviation of the per-mouse minimum %huCD45^+^,
- min %huCD45^+^ T/C value
- %huCD45^+^ *p*-value, computed using Wilcoxon rank sum test,
- Fold change in %huCD45^+^ from baseline to minimum post treatment (medium plus interquartile range)
- One column for each category of objective response (i.e. PD, PD2, etc), showing the number of mice in each category,
- Resp rate, the response rate, defined as the percentage of mice having PR or better, and

Med resp, the median response evaluation.

In addition, the *objective response* category definitions for the single mouse trial (SMT) study are as follows:

- PD when huCD45 never < 1% during study period and mouse reaches event (huCD45 >25%) at some point during the study period,
- PD1 when PD, and the mouse’s time to event ≤ 14 days post treatment initiation,
- PD2 when PD, but the mouse’s time-to-event >14 days post treatment initiation,
- SD when huCD45 never <1% and mouse never reaches event during the study period (42 days),
- PR when huCD45 <1% at least once during the study period, but not CR,
- CR when huCD45 <1% for at least 2 consecutive weekly readings during the study period, regardless of whether event is reached at a later time-point, and
- MCR when huCD45 <1% for at least 3 consecutive weekly readings at any time after treatment has been completed.

**SUPPLEMENTARY FIGURES**


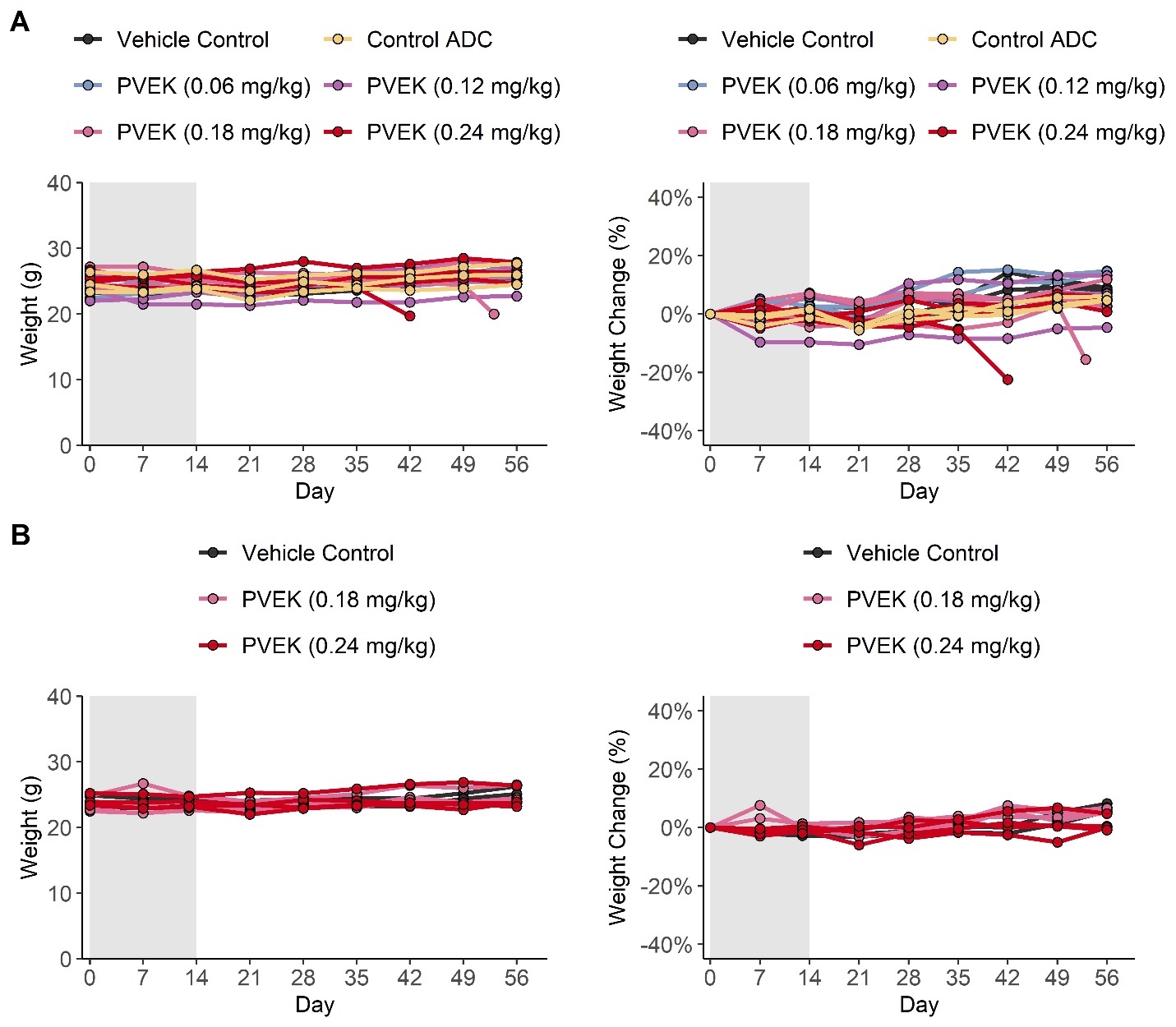


**Supplementary Figure S1. Tolerability of pivekimab sunirine (PVEK) in NSG mice.** PVEK was administered via intravenous injection once a week for three weeks, at varying doses ranging from 0.06 mg/kg to the recommended maximum tolerated dose of 0.24 mg/kg. (A) Initial tolerability testing displaying weight (left) and the % weight change (right). (B) A second tolerability study was undertaken treating mice with only the two highest doses. Displayed as weight (left) and % weight change (right). The shaded area represents the treatment window.


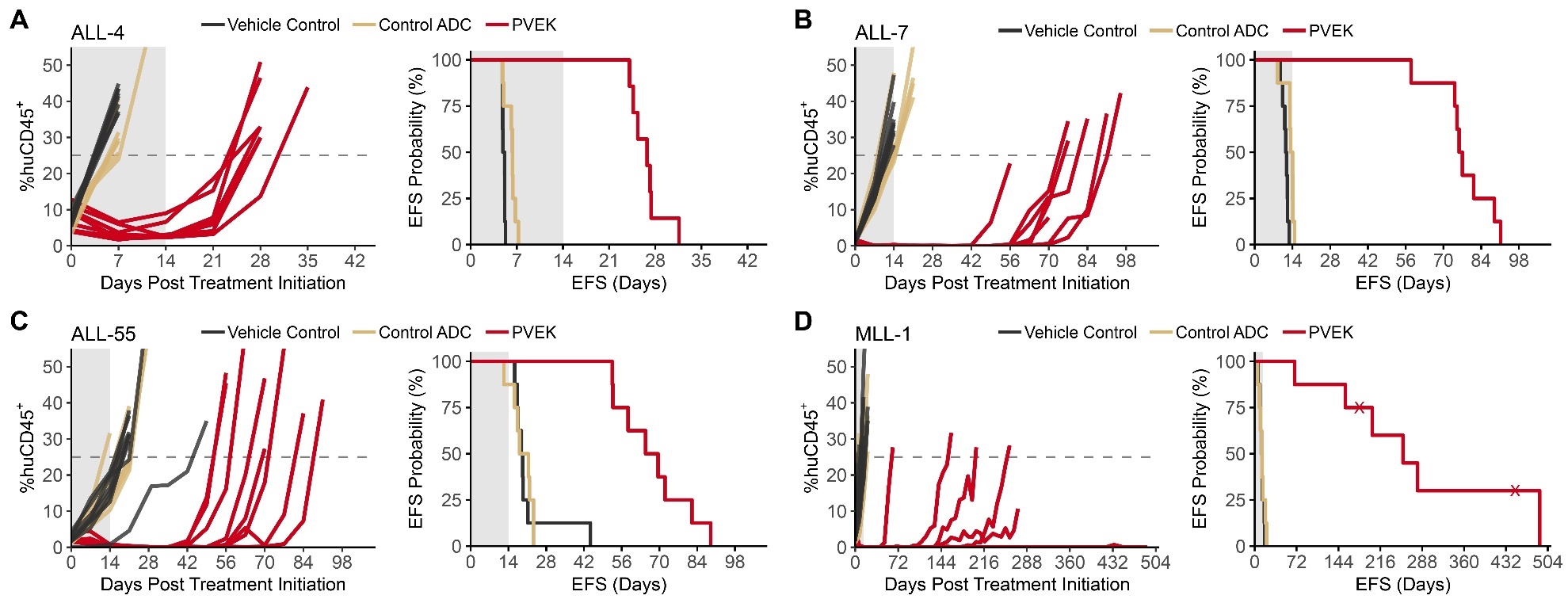


**Supplementary Figure S2.** ***In vivo* PVEK responses of pediatric ALL PDXs ALL-4, ALL-7, ALL-55 and MLL-1 in the conventional study.** Representative engraftment (left) and survival (right) plots are shown for ALL-4 (A), ALL-7 (B), ALL-55 (C), and MLL-1 (D). Leukemia burden was measured by enumeration of human CD45^+^ (%huCD45^+^) cells in the peripheral blood. PVEK treatment commenced once the %huCD45^+^ was ≥1%. EFS, event-free survival; ADC, antibody-drug conjugate; black lines, vehicle treated; gold lines, control ADC treated; red lines, PVEK treated; shaded area, treatment period; dashed line, event threshold; X, censored data.


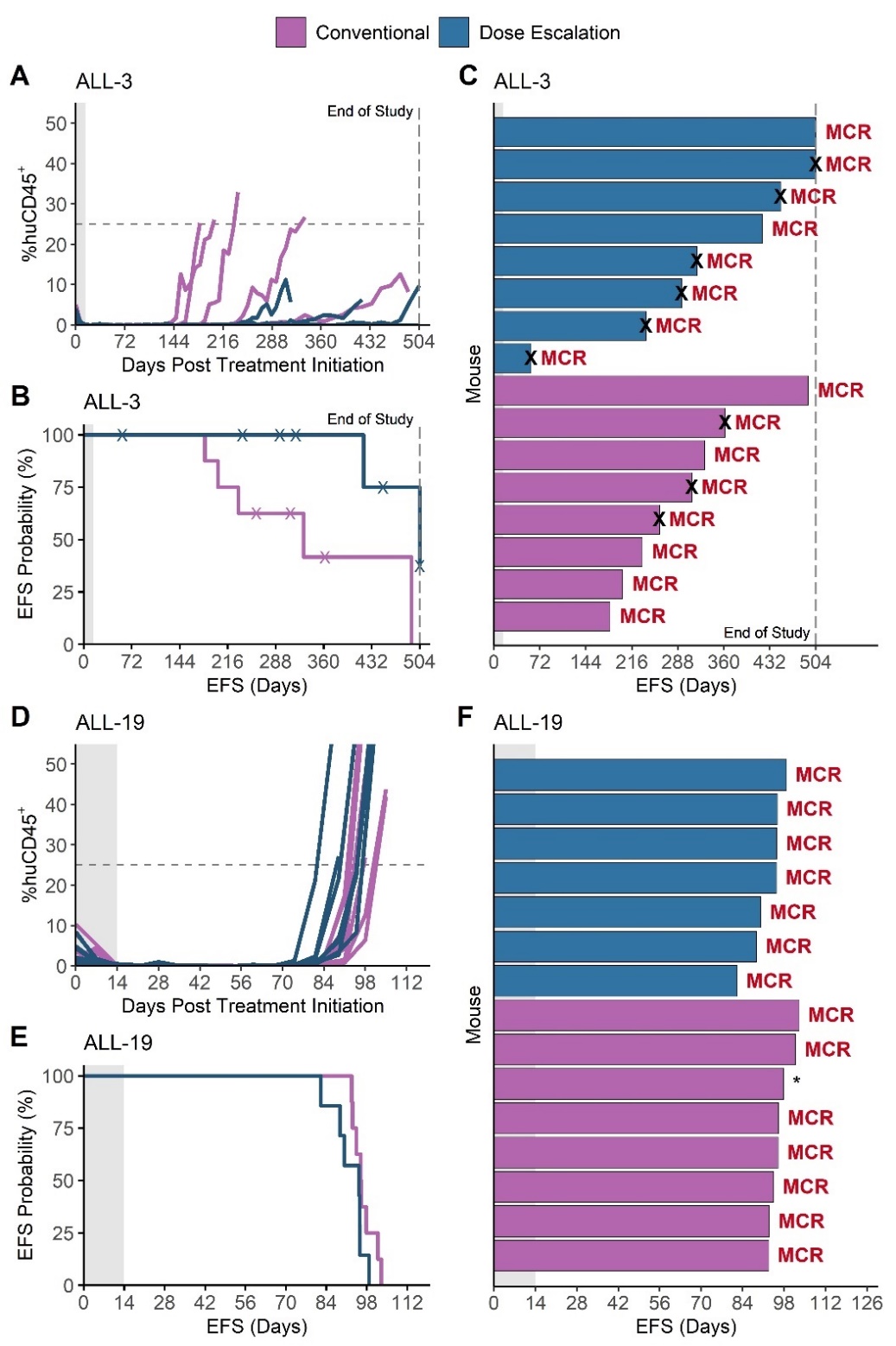


**Supplementary Figure S3. Comparison of PVEK efficacy between the conventional and dose escalation studies.** Includes results of ALL-3 and ALL-19 treated with 0.24 mg/kg PVEK. Leukemia burden was measured by enumeration of human CD45^+^ (%huCD45^+^) cells in the peripheral blood. Engraftment (A, D), survival (B, E) and swimmer (C, F) plots for ALL-3 and ALL-19, respectively. Each line/bar on engraftment (A, D) and swimmer plots (C, F) represents one mouse. EFS, event-free survival; shaded area, treatment period; horizontal dashed line, event threshold; vertical dashed line, end of study; X, censored data; MCR, maintained complete response; *, ORM not determined due to low %huCD45^+^ at treatment initiation.


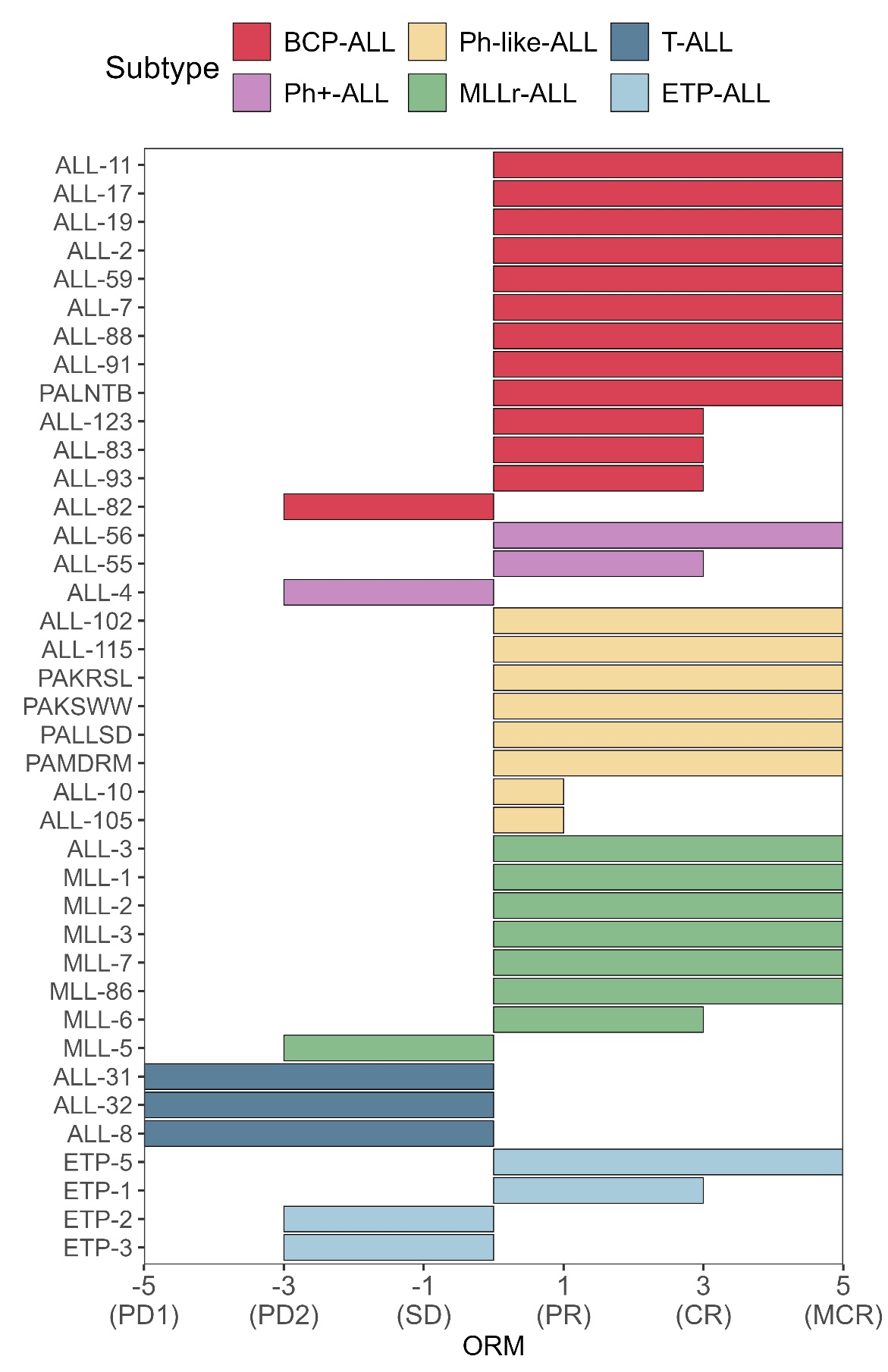


**Supplementary Figure S4. COMPARE-like plot showing ORMs of PDXs in the SMT study.** ORM scores were assigned a value from -5 (PD1) to 5 (MCR), where a positive value translates to an objective response.


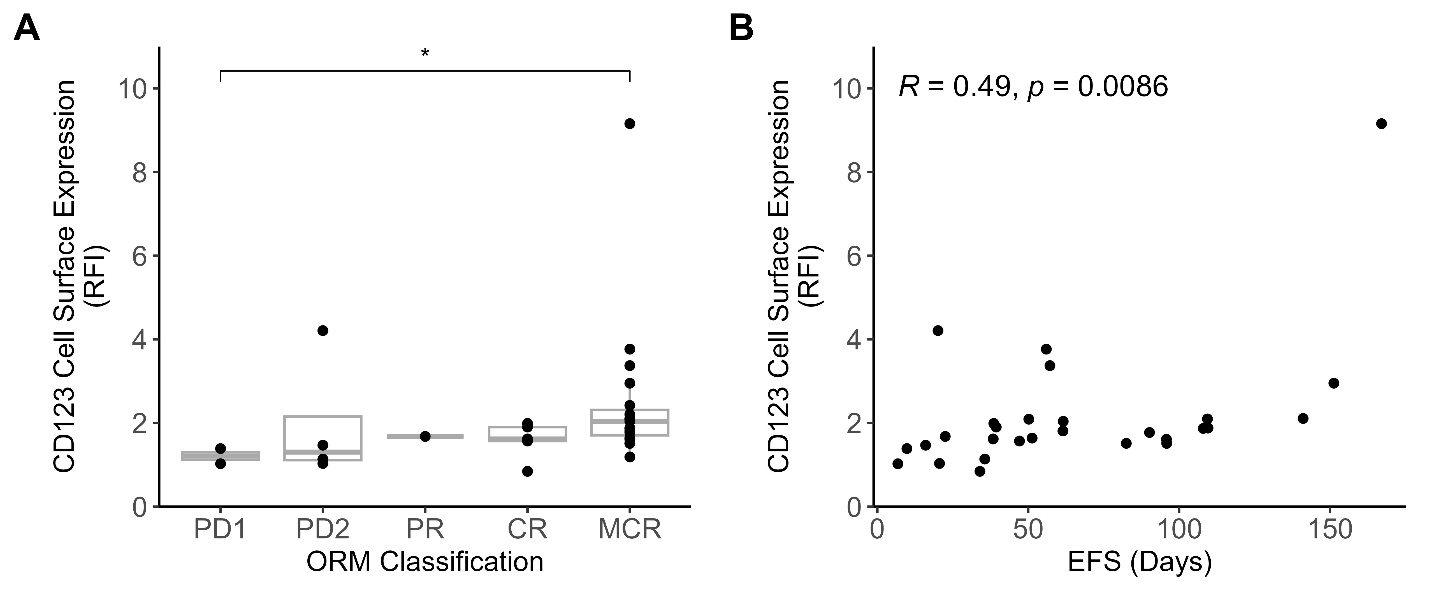


**Supplementary Figure S5. Correlation of CD123 cell surface expression and PVEK efficacy in the SMT.** CD123 cell surface expression was measured by flow cytometry and shown as the relative fluorescence intensity (RFI), calculated as the ratio of the antigen mean fluorescence intensity (MFI) and the isotype control MFI. (A) CD123 RFI for PDXs separated by their ORM classification from the SMT (n=31). (B) Pearson correlation of EFS and CD123 RFI (n=28).


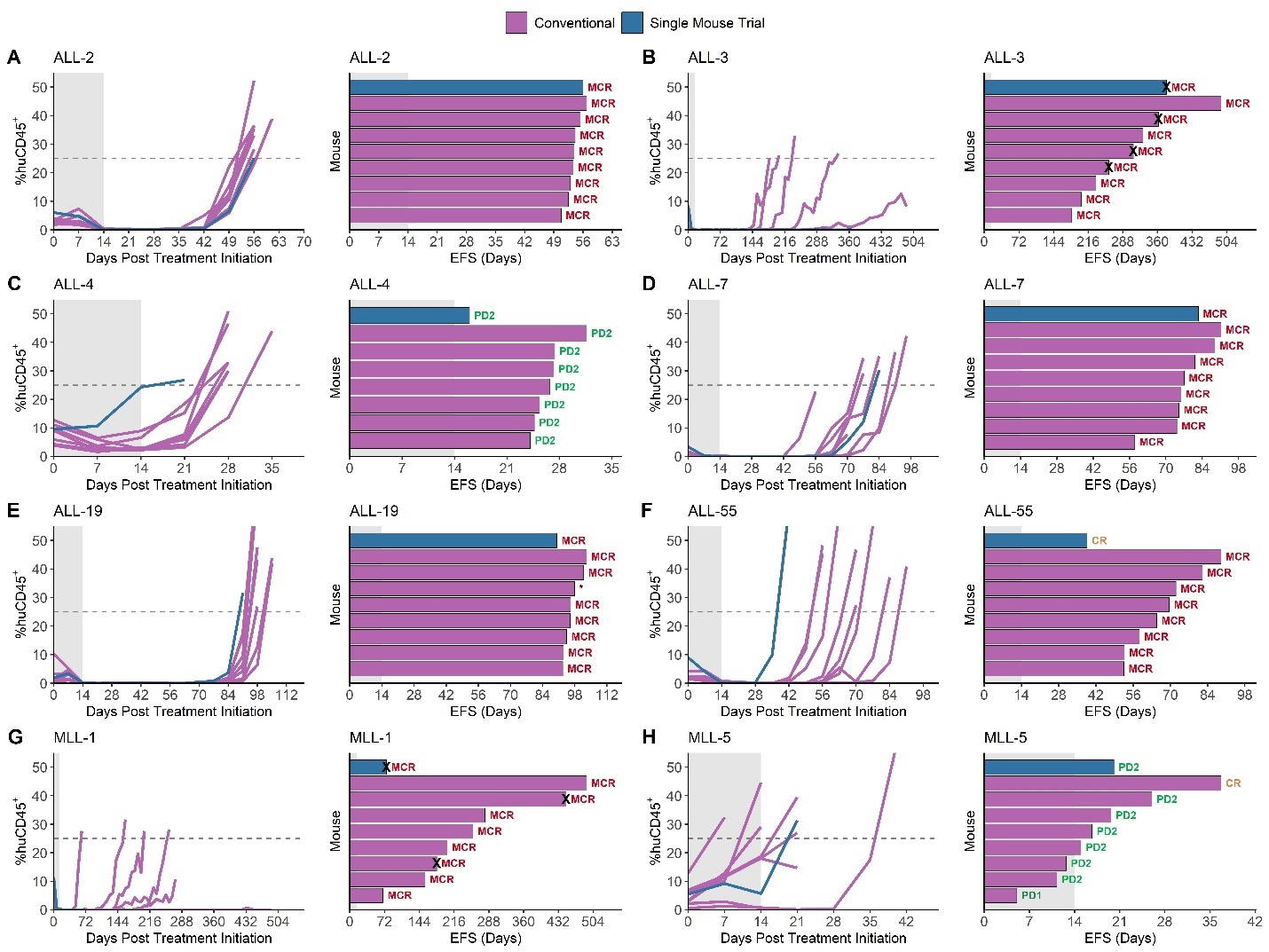


**Supplementary Figure S6. Comparison of PVEK efficacy between the conventional and SMT studies.** Leukemia burden was measured by enumeration of human CD45^+^ (%huCD45^+^) cells in the peripheral blood. Engraftment (left) and swimmer (right) plots are shown for ALL-2 (A), ALL-3 (B), ALL-4 (C), ALL-7 (D), ALL-19 (E), ALL-55 (F), MLL-1 (G) and MLL-5 (H). EFS, event-free survival; shaded area, treatment period; dashed line, event threshold; X, censored data; PD2, progressive disease 2; CR, complete response; MCR, maintained complete response; *, ORM not determined due to low %huCD45^+^ at treatment initiation.

**Supplementary Figure S7. *In vitro* cytotoxicity of the PVEK payload DGN549.** ALL PDXs ALL-2, ALL-7, ALL-19, ALL-4, ALL-55, ALL-3, MLL-1 and MLL-5 were treated with DGN549 at concentrations ranging from 0.1 pM to 1 µM and normalized to an equivalent concentration of ethanol as a control.

**SUPPLEMENTARY TABLES**

**Supplementary Table S1. Patient demographics and key molecular characteristics of the 40 ALL PDXs utilized throughout the study.**

| PDX | ALL Subtype | Age at Diagnosis (years) | Sex | Disease Status at Biopsy | Structural Variants | Copy Number Variations | Single Nucleotide Variants, Gene (amino acid change, variant allele frequency) |
| --- | --- | --- | --- | --- | --- | --- | --- |
| ALL-2 | BCP-ALL | 5.4 | F | Relapse | - | CDKN2A (HOMDEL), CDKN2B (HOMDEL) | FLT3 (Y572S, 0.62); LZTR1 (F258fs, 0.45); KMT2D (G2863fs, 0.27); KMT2D (G2863fs, 0.45); PTPRC (H1241Y, 0.25); NT5C2 (R238W, 0.45); CREBBP (D1481H, 0.59); MLLT10 (P253S, 0.71); TSPYL2 (P686A, 0.46) |
| ALL-7 | BCP-ALL | 7.3 | M | Relapse | TCF3::HLF | - | FYN (T78R, 0.41); KRAS (G12V, 0.43); EYS (S1490L, 0.56) |
| ALL-11 | BCP-ALL | 3.1 | F | Diagnosis | ETV6::RUNX1; RUNX1::LOH12CR1; PAX5::ZCCHC7; RAG1::C11orf74 | - | SMARCA4 (L1501H, 0.56); WRN (T9A, 0.54); MAPK14 (P242fs, 0.26); HGF (I142M, 0.45); NOTCH4 (L73F, 0.49); MGA (Q2484R, 0.52) |
| ALL-17 | BCP-ALL | 8.9 | F | Diagnosis | AC020743.3::IKZF1; RUNX1::ERG | - | MLLT4 (F276S, 0.25); NRAS (G12D, 0.94); RUNX1 (Q397R, 0.6) |
| ALL-19 | BCP-ALL | 16.1 | M | Relapse | NUP214::ABL1 | CDKN2A (HOMDEL), IKZF1 (HOMDEL) | MLLT4 (F276S, 0.25); NRAS (G12D, 0.94); RUNX1 (Q397R, 0.6) |
| ALL-59 | BCP-ALL | 13.5 | F | Diagnosis | ERG SV | - | CREBBP (A2219V, 0.43); SMYD3 (R66C, 0.45); CREBBP (A1671V, 0.4); LZTR1 (R283Q, 0.41); RB1 (P55L, 0.48); PRKCB (E318D, 0.52);CTCF (R339W, 0.54); NRAS (G12S, 0.99); CROCC (N598K, 0.2) |
| ALL-82 | BCP-ALL | 5.2 | F | Diagnosis | ETV6::RUNX1; ERG::EZH2 | CREBBP (AMP), MEF2C (AMP) | ROS1 (K832R, 0.21); TP53 (F212fs, 1) |
| ALL-83 | BCP-ALL | 5.2 | F | Relapse | ETV6::RUNX1;  ERG SV | MEF2C (GAIN) | FLG (H1886N, 0.27); TP53 (F212fs, 1); DDR2 (R31H, 0.26);NTRK3 (P285S, 0.51) |
| ALL-88 | BCP-ALL | 10.7 | M | Relapse | PAX5::JAK2; LRP5L::IKZF1 | CDKN2A (HOMDEL), CDKN2B (HOMDEL) | GNB1 (I80N, 0.83); NTRK2 (D47H, 0.25); NT5C2 (R39Q, 0.3);PRKCQ (R191Q, 0.35); NIPBL (Q125H, 0.4) |
| ALL-91 | BCP-ALL | 2.7 | M | Relapse | - | - | SHH (A393V, 0.54); ZFHX3 (VAAAAA777del, 0.43); NOTCH2 (A2108V, 0.43); DROSHA (D490G, 0.47); HSP90B1 (K754M, 0.52) |
| ALL-93 | BCP-ALL | 12 | M | Relapse | IKZF1::COBL | CDKN2A (HOMDEL), CDKN2B (HOMDEL) | ASXL2 (R614*, 0.39); TET2 (M1028T, 0.36); CNOT3 (R181C, 0.3); ETV6 (S203fs, 0.32); NUP98 (N314K, 0.19); LMO2 (L79R, 0.3); JAK2 (R683G, 0.44); ABL2 (K682R, 0.44); WHSC1 (G1246S, 0.46); CTNNB1 (E182G, 0.46); TSC1 (Q550E, 0.47); KRAS (G13D, 0.48); NCOR1 (S804N, 0.51); FAM49A (R63*, 0.34); LYN (, 0.37); LYN (R360Q, 0.43); EYS (V603D, 0.44); DDX31 (S442R, 0.53) |
| ALL-123 | BCP-ALL | 2.4 | F | Relapse | ETV6::RUNX1;  ERG SV | - | NRXN1 (A101V, 0.46); DHX15 (A610T, 0.43); DLL1 (T313I, 0.51); WDR64 (R1003K, 0.43); TYRO3 (Y686H, 0.17); KRAS (D119H, 0.36); SHH (R123W, 0.45); MLLT1 (V7I, 0.46); CTCF (L309P, 0.51); RET (I927M, 0.4); PIK3CA (D589H, 0.41); SF3B1 (P780L, 0.43); KIAA1549 (R1563W, 0.44); NOTCH1 (R1926H, 0.45); JAK3 (E252K, 0.48); WHSC1 (E1099K, 0.48); NUP98 (A1462V, 0.49); SETD2 (Q1301*, 0.51); TERT (R155H, 0.54); DLL1 (G500D, 0.37); UBA2 (S323*, 0.42); CDK2 (D258N, 0.47); BRD2 (D161N, 0.47) |
| PALNTB | BCP-ALL | 8.7 | F | Diagnosis | IKZF1::IKZF1; IGH::CRLF2 | CDKN2A (HOMDEL), CDKN2B (HOMDEL) | KRAS (G12V, 0.44); PAX5 (NPGLLG362fs, 0.74); PAX5 (N362fs, 0.86); PAX5 (PGLLG363fs, 1); MYB (A699T, 0.32); JAK2 (P933R, 0.47); ROS1 (, 0.49) |
| ALL-3 | MLLr-ALL | 12.9 | F | Diagnosis | KMT2A::MLLT1; PAX5::ZCCHC7 | - | CTCF (A648T, 0.53); ALK (A17T, 0.47); ALK (S15Y, 0.44); BAZ1A (R1480H, 0.47); KMT2D (G1384D, 0.48); KMT2D (G1384S, 0.49);MGA (E2760K, 0.54) |
| MLL-1 | MLLr-ALL | 0.9 | F | Diagnosis | KMT2A::EPS15 | - | COL1A1 (V349I, 0.47); KRAS (G12S, 0.4); FLG (A3799T, 0.5) |
| MLL-2 | MLLr-ALL | 0.9 | M | Diagnosis | KMT2A::EPS15 | - | EYS (TQ3100fs, 0.45); TCF3 (T12A, 0.42); MGA (K1086N, 0.39) |
| MLL-3 | MLLr-ALL | 0.9 | M | Diagnosis | KMT2A::GAS7 | - | RET (D567N, 0.49); MLLT4 (R1361Q, 0.4); SIRPA (V90I, 0.43);BCORL1 (E1693K, 0.99); CROCC (R1887Q, 0.47) |
| MLL-5 | MLLr-ALL | 0.9 | M | Diagnosis | KMT2A::MLLT10; PAX5::ZCCHC7 | - | - |
| MLL-6 | MLLr-ALL | 0.9 | M | Diagnosis | KMT2A::MLLT1; MEF2D::PTMA | - | FBN2 (H2694Q, 0.51); BRCA2 (N2135del, 0.48); CROCC (G1262R, 0.48); PHLPP1 (K1577E, 0.52) |
| MLL-7 | MLLr-ALL | 0.9 | M | Diagnosis | KMT2A::AFF1; MEF2D::PTMA; KMT2A::PTPRC | - | FLG (E3292G, 0.5); FLT3 (I836del, 0.46); MET (L575I, 0.47);CHEK1 (I465T, 0.54); CSF1R (S422C, 0.54) |
| MLL-8 | MLLr-ALL | 0.9 | F | Diagnosis | KMT2A::MLLT1 | - | KMT2D (P1964R, 0.46); KIAA1549 (G1633S, 0.15); EPHA3 (L10F, 0.34); CREBBP (H854P, 0.57); SMC3 (S39R, 0.19); KMT2E (P1545L, 0.23); MTAP (R252Q, 0.52) |
| MLL-86 | MLLr-ALL | 0.3 | F | Relapse | KMT2A::AFF1 | FLG (AMP), MEF2D (AMP) | GIGYF2 (Q1206R, 0.18); PAX5 (G290fs, 0.45); WRN (P551L, 0.42); PAX5 (P258A, 0.44); NRAS (A146T, 0.48); NUTM1 (W296R, 0.49); AMER1 (F545L, 0.49); FBN2 (E1144K, 0.44) |
| ALL-4 | Ph+-ALL | 8.7 | M | Diagnosis | BCR::ABL1 | IKZF1 (DEL) | MLLT10 (A846G, 0.58); DDX3X (T323N, 0.51); KBTBD4 (E207fs, 0.38); BCR (E552G, 0.3); FGF2 (N113S, 0.55) |
| ALL-55 | Ph+-ALL | 14.5 | M | Diagnosis | BCR::ABL1 | - | LRIG1 (Q1053L, 0.43); CTNNB1 (N287S, 0.48); KMT2D (T4350A, 0.47); ABL2 (G896R, 0.63); GIGYF2 (E875del, 0.47) |
| ALL-56 | Ph+-ALL | 10 | M | Diagnosis | BCR::ABL1 | - | - |
| ALL-10 | Ph-like ALL | 4 | M | Diagnosis | P2RY8::CRLF2 | CDKN2A (HOMDEL), CDKN2B (HOMDEL) | KMT2D (P886fs, 0.29); JAK1 (V658L, 0.56); HDAC7 (P145fs, 0.47) |
| ALL-102 | Ph-like ALL | 3.1 | M | Diagnosis | P2RY8::CRLF2; RAG1::C11orf74 | IKZF1 (DEL) | KMT2D (T1246M, 0.44); JAK2 (T875N, 0.41) |
| ALL-105 | Ph-like ALL | 3.1 | M | Relapse | P2RY8::CRLF2; RAG1::C11orf74 | IKZF1 (DEL) | KMT2D (T1246M, 0.38) |
| ALL-115 | Ph-like ALL | 3.1 | M | Relapse | P2RY8::CRLF2; RAG1::C11orf74 | - | JAK2 (R683T, 0.26); KMT2D (T1246M, 0.47); TP53 (M246I, 0.37); CHEK1 (D258N, 0.41); DROSHA (E1193*, 0.55); NR3C2 (Q61*, 0.4) |
| PAKRSL | Ph-like ALL | 13.7 | F | Diagnosis | CRLF2::IGH; CCM2::IKZF1 | CDKN2A (HOMDEL), CDKN2B (HOMDEL) | JAK2 (R683S, 0.6); NRAS (G12D, 0.44); TET2 (G126R, 0.47); BCOR (D1631N, 0.67); EYS (C685G, 0.43); GAK (R1165H, 0.48);PHLPP1 (D346E, 0.52) |
| PAKSWW | Ph-like ALL | 15.1 | M | Diagnosis | - | IKZF1 (DEL) | KRAS (G12V, 0.47); PIK3CG (R273H, 0.2); TP53 (R248Q, 0.99);TSC1 (P56L, 0.45); SMC1A (R57W, 0.99) |
| PALLSD | Ph-like ALL | 14.2 | M | Diagnosis | IKZF1::IKZF1 | CDKN2A (HOMDEL), CDKN2B (HOMDEL), PAX5 (DEL) | TSC2 (R1122H, 0.46); BCR (Q1251R, 0.34); JAK2 (R683G, 0.42);JAK1 (R724C, 0.51); GNB1 (G116V, 0.46) |
| PAMDRM | Ph-like ALL | 7.9 | M | Diagnosis | RAG1::C11orf74; IGH::CRLF2 | CDKN2A (HOMDEL), CDKN2B (HOMDEL), IKZF1 (DEL), EBF1 (DEL) | MGA (G2634R, 0.43); ETV6 (Y391C, 0.43); IKZF3 (T190M, 0.49); KMT2D (696_705SPTSPPPEDS>S, 0.28); PAX5 (A322fs, 0.44); JAK2 (681_682insGP, 0.67); NRAS (Q61H, 0.33); MTOR (T1876I, 0.39); ERBB3 (A1030T, 0.39); TSC1 (P641S, 0.45); FGFR4 (V746D, 0.46); MSH6 (G1157S, 0.5); CCND3 (S169F, 0.52); PDGFRB (S373L, 0.53); COL1A1 (P146T, 0.53); HDAC7 (K167fs, 0.55); SHANK2 (R1164C, 0.42);LEMD3 (S894F, 0.48) |
| ALL-8 | T-ALL | 12.6 | M | Relapse | - | CDKN2A (HOMDEL), CDKN2B (HOMDEL) | FAT2 (R2678*, 0.43); NT5C2 (R367Q, 0.56); FBXW7 (R465C, 0.42); ASXL1 (D863G, 0.47); SMARCA4 (R1189Q, 0.49); IL1B (R75K, 0.47); FBN2 (D1008H, 0.51) |
| ALL-31 | T-ALL | 10.1 | M | Diagnosis | PTMA::NPM1; LMO1::TRBC1; LMO1::TRBC2 | CDKN2A (HOMDEL), CDKN2B (HOMDEL) | NRG2 (A792T, 0.49); MLLT4 (R915H, 0.47) |
| ALL-32 | T-ALL | 11.1 | M | Relapse | NUP214::ABL1 | CDKN2A (HOMDEL), CDKN2B (HOMDEL) | ATF7IP (H115R, 0.48); ABL1 (E255K, 0.37); NOTCH2 (P2087T, 0.42); CTCF (, 0.45); NOTCH1 (L1593P, 0.47); WHSC1 (E1099K, 0.52);MTOR (A461V, 0.59); FGF3 (R145Q, 0.48) |
| ETP-1 | ETP-ALL | 16 | M | Diagnosis | ATRX::MIR325HG; TP53::SNX31 | - | NOTCH1 (S2492*, 0.44); RUNX1 (A122fs, 0.47); NUP214 (A1376fs, 0.39); PHF6 (N147fs, 0.92); FGFR2 (E586Q, 0.43); ZFHX3 (G3511S, 0.51); EZH2 (S695L, 0.97); MED12 (R516H, 1); ZFP36L2 (A348T, 0.48); GNB1 (D76G, 0.51) |
| ETP-2 | ETP-ALL | 3 | M | Diagnosis | HLA-A::HLA-B; SET::PTMA | - | FAT2 (N731K, 0.39); MLLT3 (156_156S>SS, 0.39); NOTCH1 (E1408G, 0.28); JAK1 (S703I, 1); IGF1R (S1281I, 0.45) |
| ETP-3 | ETP-ALL | 14 | M | Diagnosis | ERG::IGJ | CDKN2A (HOMDEL), CDKN2B (HOMDEL) | GATA3 (TSTPLW270fs, 0.27); GATA3 (STPLW271fs, 0.32); NOTCH1 (PEQ1582del, 0.34); NOTCH1 (P1582fs, 0.52); NRXN1 (L607W, 0.51); FBN2 (I1248V, 0.49); NOTCH1 (Q1584fs, 0.5); TERT (A67P, 0.34); NOTCH1 (P1582T, 0.57); GATA3 (N286T, 0.69); JAK1 (S703I, 1); SOS1 (R982Q, 0.38); GIGYF2 (R407Q, 0.38); NOTCH4 (T684M, 0.45) |
| ETP-5 | ETP-ALL | 15.9 | M | Diagnosis | KMT2A::MLLT4; KMT2A::AFDN | - | TERT (A67P, 0.35); BRCA2 (M927V, 0.38); FLT3 (D835Y, 0.43); KIT (D908E, 0.46); PTPN11 (A72V, 0.48); H3F3A (K37R, 0.49); CDH4 (R204W, 0.43); NOTCH4 (N1926S, 0.49) |

**Supplementary Table S2.** **Cell seeding densities for PDXs in the *ex vivo* DGN549 cytotoxicity assays.**

| PDX | Seeding Density (cells per mL) |
| --- | --- |
| ALL-2 | 2.5 × 10^6^ |
| ALL-3 | 4 × 10^6^ |
| ALL-4 | 3 × 10^6^ |
| ALL-7 | 2 × 10^6^ |
| ALL-19 | 2 × 10^6^ |
| ALL-55 | 2 × 10^6^ |
| MLL-1 | 3.5 × 10^6^ |
| MLL-5 | 1.5 × 10^6^ |

**Supplementary Table S3. Hematology results of the first PVEK tolerability study.** Values represent the median of weekly hematology results for each group throughout the study (n=3 for vehicle control and PVEK [0.06 mg/kg] groups; n=4 for all other groups). The reference range was determined by analysis of 307 hematology measurements from 71 untreated NSG mice, mean ± 2SD. Underlined values lie outside the reference range.

|  | Vehicle Control | | | | Control ADC | | | | PVEK  (0.06 mg/kg) | | | | PVEK  (0.12 mg/kg) | | | | PVEK  (0.18 mg/kg) | | | | PVEK  (0.24 mg/kg) | | | | Reference Range |
| --- | --- | --- | --- | --- | --- | --- | --- | --- | --- | --- | --- | --- | --- | --- | --- | --- | --- | --- | --- | --- | --- | --- | --- | --- | --- |
|  | Wk2 | Wk3 | Wk4 | Wk5 | Wk2 | Wk3 | Wk4 | Wk5 | Wk2 | Wk3 | Wk4 | Wk5 | Wk2 | Wk3 | Wk4 | Wk5 | Wk2 | Wk3 | Wk4 | Wk5 | Wk2 | Wk3 | Wk4 | Wk5 |  |
| RBC (x10^6^/µL) | 10.2 | 8.8 | 10.0 | 9.7 | 9.8 | 10.5 | 9.3 | 10.6 | 9.6 | 9.2 | 9.5 | ND | 10.0 | 9.8 | 9.0 | ND | 9.7 | 10.4 | 9.1 | 9.7 | 9.6 | 9.9 | 9.7 | 10.3 | 6.4-11.8 |
| HGB (g/L) | 170 | 146 | 164 | 160 | 160 | 168 | 152 | 168 | 162 | 156 | 158 | ND | 168 | 164 | 149 | ND | 162 | 171 | 153 | 158 | 163 | 169 | 161 | 170 | 115-192 |
| HCT (L/L) | 0.5 | 0.5 | 0.5 | 0.5 | 0.5 | 0.5 | 0.5 | 0.5 | 0.5 | 0.5 | 0.5 | ND | 0.5 | 0.5 | 0.5 | ND | 0.5 | 0.5 | 0.5 | 0.5 | 0.5 | 0.5 | 0.5 | 0.5 | 0.4-0.6 |
| PLT  (x10^3^/µL) | 1508 | 1288 | 1556 | 1600 | 1596 | 1640 | 1730 | 1876 | 1274 | 1432 | 1586 | ND | 1383 | 1577 | 1545 | ND | 1432 | 1674 | 1518 | 1819 | 1522 | 1660 | 1532 | 1661 | 518-1753 |
| WBC (x10^3^/µL) | 5.0 | 4.0 | 3.2 | 3.4 | 2.6 | 3.8 | 2.8 | 5.0 | 3.2 | 2.8 | 2.4 | ND | 4.3 | 4.8 | 2.3 | ND | 2.5 | 2.9 | 2.1 | 3.3 | 2.4 | 3.2 | 2.2 | 2.8 | 0.3-6.7 |

PVEK, pivekimab sunirine; Wk, week of the study; RBC, Red Blood Cells; HGB, Hemoglobin; HCT, Hematocrit; PLT, Platelets; WBC, White Blood Cells.

**Supplementary Table S4. Blood biochemistry results of the first PVEK tolerability study.** Blood was pooled from n = 3 mice (vehicle control, control ADC and 0.06 mg/kg PVEK groups; one analysis per group) or n = 4 mice (0.12 [one analysis] 0.18 and 0.24 mg/kg groups; two analyses per group). The reference range was determined by analysis of biochemistry measurements from 71 untreated NSG mice, mean ± 2SD. Data are presented as the median for each group. Underlined values lie outside the reference range.

|  | Vehicle Control | | | | Control ADC | | | | PVEK  (0.06 mg/kg) | | | | PVEK  (0.12 mg/kg) | | | | PVEK  (0.18 mg/kg) | | | | PVEK  (0.24 mg/kg) | | | | Reference Range |
| --- | --- | --- | --- | --- | --- | --- | --- | --- | --- | --- | --- | --- | --- | --- | --- | --- | --- | --- | --- | --- | --- | --- | --- | --- | --- |
|  | Wk2 | Wk3 | Wk4 | Wk5 | Wk2 | Wk3 | Wk4 | Wk5 | Wk2 | Wk3 | Wk4 | Wk5 | Wk2 | Wk3 | Wk4 | Wk5 | Wk2 | Wk3 | Wk4 | Wk5 | Wk2 | Wk3 | Wk4 | Wk5 |  |
| ALB (g/L) | 42.0 | 42.0 | 45.0 | 44.0 | ND | 41.0 | 42.0 | 42.0 | 40.0 | 41.0 | 44.0 | ND | 42.0 | 41.0 | 43.0 | ND | 40.5 | 42.0 | 43.5 | 43.0 | 43.5 | 38.0 | 45.5 | 44.5 | 21.9-59.9 |
| ALP (U/L) | 44.0 | 71.0 | 78.0 | 89.0 | ND | 60.0 | 69.0 | 58.0 | 76.0 | 72.0 | 64.0 | ND | 52.0 | 52.0 | 77.0 | ND | 41.5 | 37.5 | 66.0 | 60.0 | 46.0 | 43.0 | 65.0 | 42.5 | 18.7-109 |
| ALT (U/L) | 38.0 | 34.0 | 27.0 | 29.0 | ND | 29.0 | 27.0 | 30.0 | 32.0 | 37.0 | 26.0 | ND | 33.0 | 44.5 | 30.0 | ND | 33.0 | 42.5 | 33.0 | 32.5 | 36.0 | 35.0 | 29.5 | 26.5 | 11.7-38.4 |
| AMY (U/L) | 770 | 936 | 893 | 868 | ND | 867 | 962 | 985 | 870 | 841 | 863 | ND | 824 | 863 | 824 | ND | 855 | 892 | 966 | 831 | 889 | 873 | 930 | 899 | 529-1226 |
| TBIL (µmol/L) | 5.0 | 5.0 | 5.0 | 6.0 | ND | 5.0 | 5.0 | 5.0 | 5.0 | 5.0 | 5.0 | ND | 4.0 | 4.5 | 5.0 | ND | 4.0 | 5.0 | 5.0 | 5.0 | 4.0 | 4.0 | 5.0 | 4.0 | 2.3-9.5 |
| BUN (mmol/L) | 7.7 | 8.9 | 7.0 | 7.5 | ND | 10.1 | 6.2 | 7.3 | 11.0 | 9.7 | 7.2 | ND | 10.2 | 11.8 | 6.5 | ND | 10.9 | 10.6 | 6.6 | 7.3 | 9.1 | 10.4 | 7.3 | 7.2 | 4.2-10.1 |
| CA (mmol/L) | 2.6 | 2.7 | 2.5 | 2.5 | ND | 2.7 | 2.5 | 2.6 | 2.5 | 2.6 | 2.6 | ND | 2.6 | 2.6 | 2.5 | ND | 2.6 | 2.7 | 2.5 | 2.5 | 2.7 | 2.7 | 2.6 | 2.6 | 1.5-3.3 |
| PHOS (mmol/L) | 2.5 | 2.6 | 1.7 | 1.9 | ND | 2.3 | 1.7 | 1.6 | 2.4 | 2.4 | 1.8 | ND | 2.4 | 2.2 | 2.2 | ND | 2.3 | 2.0 | 2.0 | 1.9 | 2.2 | 1.8 | 1.7 | 1.5 | 0.8-3.4 |
| CRE (mmol/L) | <18 | <18 | <18 | 38.0 | ND | 37.0 | <18 | <18 | <18 | 30.0 | 19.0 | ND | <18 | 25.0 | <18 | ND | <18 | 36.0 | <18 | 25.0 | 25.0 | <18 | 30.0 | 27.0 | 8.1-34.7 |
| GLU (mmol/L) | 6.5 | 7.6 | 9.0 | 7.7 | ND | 8.7 | 5.3 | 7.0 | 8.6 | 6.1 | 8.0 | ND | 7.4 | 7.2 | 6.5 | ND | 7.1 | 5.6 | 6.2 | 7.8 | 4.4 | 4.8 | 6.0 | 6.5 | 2.3-11.2 |
| Na^+^ (mmol/L) | 139 | 148 | 143 | 143 | ND | 147 | 141 | 142 | 139 | 145 | 141 | ND | 142 | 141 | 140 | ND | 140 | 146 | 141 | 143 | 144 | 146 | 142 | 142 | 139-166 |
| K^+^ (mmol/L) | >8.5 | >8.5 | 7.9 | 8.1 | ND | 8.3 | 7.7 | 8.4 | 8.0 | 8.5 | 8.0 | ND | >8.5 | 7.7 | 7.7 | ND | 8.5 | 8.5 | 7.8 | 8.1 | >8.5 | >8.5 | 8.3 | >8.5 | 5.8-8.8 |
| TP  (g/L) | 55.0 | 55.0 | 58.0 | 60.0 | ND | 54.0 | 59.0 | 61.0 | 51.0 | 55.0 | 58.0 | ND | 54.0 | 52.5 | 56.0 | ND | 52.5 | 56.5 | 58.5 | 56.0 | 57.0 | 57.0 | 60.5 | 59.0 | 35.5-69.0 |
| GLOB (g/L) | 13.0 | 13.0 | 13.0 | 16.0 | ND | 12.0 | 17.0 | 18.0 | 11.0 | 13.0 | 14.0 | ND | 12.0 | 12.0 | 13.0 | ND | 12.0 | 13.5 | 15.0 | 13.0 | 13.5 | 19.0 | 15.0 | 14.0 | 6.6-19.2 |

Wk, week of the study; ALB, Albumin; ALP, Alkaline Phosphatase; ALT, Alanine Aminotransferase; AMY, Amylase; TBIL, Total Bilirubin; BUN, Blood Urea Nitrogen; CA, Calcium; PHOS, Phosphate; CRE, Creatinine; GLU, Glucose; Na+, Sodium; K+, Potassium; TP, Total Protein; GLOB, Globulin; ND, no data.

**Supplementary Table S5.** **Hematology results of the second PVEK tolerability study.** Values represent the median of weekly hematology results for each group throughout the study (n=2 for vehicle control group; n=4 for all other groups). The reference range was determined by analysis of 307 hematology measurements from 71 untreated NSG mice, mean ± 2SD.

|  | Vehicle Control | | | | PVEK (0.18 mg/kg) | | | | PVEK (0.24 mg/kg) | | | | Reference Range |
| --- | --- | --- | --- | --- | --- | --- | --- | --- | --- | --- | --- | --- | --- |
|  | Wk2 | Wk3 | Wk4 | Wk5 | Wk2 | Wk3 | Wk4 | Wk5 | Wk2 | Wk3 | Wk4 | Wk5 |  |
| RBC (x10^6/µL) | 9.6 | 9.4 | 10.0 | 9.1 | 9.1 | 9.4 | 9.4 | 8.9 | 9.2 | 9.2 | 9.9 | 9.6 | 6.4-11.8 |
| HGB (g/L) | 156 | 151 | 162 | 150 | 149 | 153 | 155 | 148 | 150 | 150 | 165 | 157 | 115-192 |
| HCT (L/L) | 0.5 | 0.5 | 0.5 | 0.5 | 0.5 | 0.5 | 0.5 | 0.4 | 0.5 | 0.5 | 0.5 | 0.5 | 0.4-0.6 |
| PLT  (x10^3/µL) | 1332 | 1330 | 1378 | 1507 | 1369 | 1494 | 1286 | 1523 | 1345 | 1533 | 1304 | 1522 | 518-1753 |
| WBC (x10^3/µL) | 2.1 | 2.0 | 2.7 | 2.7 | 2.3 | 2.4 | 2.6 | 1.8 | 2.2 | 2.5 | 3.4 | 1.6 | 0.3-6.7 |

Wk, week of the study; RBC, Red Blood Cells; HGB, Hemoglobin; HCT, Hematocrit; PLT, Platelets; WBC, White Blood Cells.

**Supplementary Table S6.** **Blood biochemistry results of the second PVEK tolerability study.** Blood was pooled from n = 2 mice (vehicle control, one analysis per group) or n = 4 mice (0.18 and 0.24 mg/kg PVEK groups; two analyses per group). The reference range was determined by analysis of biochemistry measurements from 71 untreated NSG mice, mean ± 2SD. Data are presented as the median for each group. Underlined values lie outside the reference range.

|  | Vehicle Control | | | | PVEK (0.18 mg/kg) | | | | PVEK (0.24 mg/kg) | | | | Reference Range |
| --- | --- | --- | --- | --- | --- | --- | --- | --- | --- | --- | --- | --- | --- |
|  | Wk2 | Wk3 | Wk4 | Wk5 | Wk2 | Wk3 | Wk4 | Wk5 | Wk2 | Wk3 | Wk4 | Wk5 |  |
| ALB (g/L) | 41.0 | 39.0 | 40.0 | 37.0 | 41.0 | 40.5 | 42.0 | 43.0 | ND | 38.5 | 42.0 | 41.0 | 21.9-59.9 |
| ALP (U/L) | 77.0 | 68.0 | 71.0 | 79.0 | 81.5 | 71.5 | 70.0 | 82.0 | ND | 79.5 | 95.0 | 83.0 | 18.7-109 |
| ALT (U/L) | 37.0 | 33.0 | 28.0 | 31.0 | 33.0 | 36.5 | 34.0 | 33.0 | ND | 43.0 | 27.0 | 33.0 | 11.7-38.4 |
| AMY (U/L) | 1021 | 1042 | 1033 | 988 | 1066 | 1065 | 1130 | 1081 | ND | 1139 | 1163 | 1056 | 529-1226 |
| TBIL (µmol/L) | 5.0 | 5.0 | 5.0 | 5.0 | 5.0 | 4.0 | 4.5 | 5.0 | ND | 5.0 | 4.0 | 5.0 | 2.3-9.5 |
| BUN (mmol/L) | 5.9 | 5.4 | 6.4 | 5.9 | 6.6 | 6.7 | 7.0 | 6.7 | ND | 6.3 | 9.2 | 7.1 | 4.2-10.1 |
| CA (mmol/L) | 2.6 | 2.5 | 2.6 | 2.5 | 2.5 | 2.6 | 2.5 | 2.5 | ND | 2.5 | 2.6 | 2.5 | 1.5-3.3 |
| PHOS (mmol/L) | 2.1 | 1.9 | 1.6 | 1.4 | 2.1 | 2.0 | 1.6 | 1.9 | ND | 2.0 | 1.6 | 1.7 | 0.8-3.4 |
| CRE (mmol/L) | <18 | <18 | <18 | 20.0 | 26.0 | 27.0 | 23.0 | 21.0 | ND | <18 | 19.0 | 20.0 | 8.1-34.7 |
| GLU (mmol/L) | 8.5 | 10.1 | 9.1 | 8.2 | 7.7 | 7.6 | 6.5 | 7.1 | ND | 6.5 | 7.4 | 6.1 | 2.3-11.2 |
| Na^+^ (mmol/L) | 145 | 145 | 148 | 148 | 145 | 151 | 81 | 150 | ND | 151 | 153 | 151 | 139-166 |
| K^+^ (mmol/L) | 8.3 | 7.1 | 7.3 | 7.0 | 7.9 | 8.1 | 7.3 | 7.3 | ND | 7.8 | 7.8 | 7.3 | 5.8-8.8 |
| TP (g/L) | 64.0 | 61.0 | 58.0 | 58.0 | 62.0 | 63.0 | 60.5 | 60.0 | ND | 59.0 | 60.0 | 60.0 | 35.5-69.0 |
| GLOB (g/L) | 23.0 | 21.0 | 19.0 | 21.0 | 21.0 | 22.5 | 18.5 | 17.0 | ND | 20.5 | 18.0 | 19.0 | 6.6-19.2 |

Wk, week of the study; ALB, Albumin; ALP, Alkaline Phosphatase; ALT, Alanine Aminotransferase; AMY, Amylase; TBIL, Total Bilirubin; BUN, Blood Urea Nitrogen; CA, Calcium; PHOS, Phosphate; CRE, Creatinine; GLU, Glucose; Na+, Sodium; K+, Potassium; TP, Total Protein; GLOB, Globulin; ND, no data.

**Supplementary Table S7. Detailed summary table for the *in vivo* conventional study with PVEK.**

|  |  |  | **Animal Counts/**  **Status** | | | | | **EFS Evaluation** | | | | | **%huCD45 evaluation** | | | | | | **Response Evaluation** | | | | | | | | | |
| --- | --- | --- | --- | --- | --- | --- | --- | --- | --- | --- | --- | --- | --- | --- | --- | --- | --- | --- | --- | --- | --- | --- | --- | --- | --- | --- | --- | --- |
| **Subtype** | **PDX** | **Treatment Group** | **N** | **Nd** | **Nx** | **Na** | **Nev** | **KM med** | **EFS T-C** | **EFS T/C** | **p-value exact log-rank** | **p-value Gehan-Wilcoxon** | **Baseline mean±SD** | **p-value** | **minCD45 mean±SD** | **minCD45 T/C** | **p- value** | **∆CD45/Baseline Median(IQR)** | **PD** | **PD1** | **PD2** | **SD** | **PR** | **CR** | **MCR** | **Excl**  **ORM** | **Response  rate** | **Med  resp** |
| BCP-ALL | ALL-2 | Vehicle Control | 8 | 0 | 0 | 8 | 8 | 13.8 |  |  |  |  | 2.513±0.751 |  | 12.900±4.874 |  |  | 4.06(3.38,5.09) | 8 |  |  | 0 | 0 | 0 | 0 | 0 | 0% | PD1 |
|  |  | Control ADC | 8 | 0 | 0 | 8 | 8 | 15.1 | 1.3 | 1.1 | p = 0.289 | p = 0.207 | 2.788±1.095 | p = 0.792 | 12.488±3.499 | 1.0 | p = 0.793 | 3.51(2.73,4.42) | 8 | 8 | 0 | 0 | 0 | 0 | 0 | 0 | 0% | PD1 |
|  |  | PVEK | 8 | 0 | 0 | 8 | 8 | 53.7 | 39.9 | 3.9 | p < 0.001 | p < 0.001 | 2.663±0.776 | p = 0.958 | 0.012±0.035 | 0.0 | p < 0.001 | -1.00(-1.00,-1.00) | 0 | 0 | 0 | 0 | 0 | 0 | 8 | 0 | 100% | MCR |
|  |  | Control ADC vs PVEK |  |  |  |  |  |  | 38.6 | 3.6 | p < 0.001 | p < 0.001 |  | p = 0.793 |  | 0.0 | p < 0.001 |  |  |  |  |  |  |  |  |  |  |  |
|  | ALL-7 | Vehicle Control | 8 | 0 | 0 | 8 | 8 | 11.7 |  |  |  |  | 1.175±0.311 |  | 14.662±1.736 |  |  | 11.55(9.86,14.93) | 8 |  |  | 0 | 0 | 0 | 0 | 0 | 0% | PD1 |
|  |  | Control ADC | 8 | 0 | 0 | 8 | 8 | 13.7 | 2 | 1.2 | p = 0.004 | p = 0.007 | 1.363±0.396 | p = 0.340 | 13.562±3.345 | 0.9 | p = 0.103 | 9.11(8.09,10.53) | 8 | 8 | 0 | 0 | 0 | 0 | 0 | 0 | 0% | PD1 |
|  |  | PVEK | 8 | 0 | 0 | 8 | 8 | 76.4 | 64.7 | 6.6 | p < 0.001 | p < 0.001 | 1.288±0.280 | p = 0.492 | 0.000±0.000 | 0.0 | p < 0.001 | -1.00(-1.00,-1.00) | 0 | 0 | 0 | 0 | 0 | 0 | 8 | 0 | 100% | MCR |
|  |  | Control ADC vs PVEK |  |  |  |  |  |  | 62.7 | 5.6 | p < 0.001 | p < 0.001 |  | p = 0.958 |  | 0.0 | p < 0.001 |  |  |  |  |  |  |  |  |  |  |  |
|  | ALL-19 | Vehicle Control | 8 | 0 | 0 | 8 | 8 | 6.3 |  |  |  |  | 4.112±7.181 |  | 32.225±15.302 |  |  | 27.94(7.68,47.71) | 8 |  |  | 0 | 0 | 0 | 0 | 0 | 0% | PD1 |
|  |  | Control ADC | 8 | 0 | 0 | 8 | 8 | 7.3 | 1.0 | 1.2 | p = 0.374 | p = 0.295 | 3.288±6.039 | p = 0.875 | 23.712±10.164 | 0.7 | p = 0.141 | 24.68(15.12,32.08) | 7 | 7 | 0 | 0 | 0 | 0 | 0 | 1 | 0% | PD1 |
|  |  | PVEK | 8 | 0 | 0 | 8 | 8 | 96.0 | 89.7 | 15.2 | p < 0.001 | p < 0.001 | 2.337±3.364 | p = 0.916 | 0.000±0.000 | 0.0 | p < 0.001 | -1.00(-1.00,-1.00) | 0 | 0 | 0 | 0 | 0 | 0 | 7 | 1 | 100% | MCR |
|  |  | Control ADC vs PVEK |  |  |  |  |  |  | 88.7 | 13.2 | p < 0.001 | p < 0.001 |  | p = 0.833 |  | 0.0 | p < 0.001 |  |  |  |  |  |  |  |  |  |  |  |
| Ph+-ALL | ALL-4 | Vehicle Control | 8 | 0 | 0 | 8 | 8 | 5.0 |  |  |  |  | 7.263±1.522 |  | 41.075±3.058 |  |  | 4.83(4.08,5.63) | 8 |  |  | 0 | 0 | 0 | 0 | 0 | 0% | PD1 |
|  |  | Control ADC | 8 | 0 | 0 | 8 | 8 | 6.3 | 1.3 | 1.3 | p = 0.010 | p = 0.016 | 7.413±2.813 | p = 0.752 | 31.025±5.833 | 0.8 | p = 0.005 | 3.79(2.21,4.80) | 8 | 8 | 0 | 0 | 0 | 0 | 0 | 0 | 0% | PD1 |
|  |  | PVEK | 8 | 0 | 0 | 8 | 7 | 26.7 | 21.7 | 5.3 | p < 0.001 | p < 0.001 | 7.775±3.360 | p = 1.000 | 3.113±1.502 | 0.1 | p < 0.001 | -0.56(-0.66,-0.48) | 7 | 0 | 7 | 0 | 0 | 0 | 0 | 1 | 0% | PD2 |
|  |  | Control ADC vs PVEK |  |  |  |  |  |  | 20.4 | 4.2 | p < 0.001 | p < 0.001 |  | p = 0.834 |  | 0.1 | p < 0.001 |  |  |  |  |  |  |  |  |  |  |  |
|  | ALL-55 | Vehicle Control | 8 | 0 | 0 | 8 | 8 | 19.0 |  |  |  |  | 2.225±1.295 |  | 7.838±4.064 |  |  | 2.85(2.08,3.58) | 7 |  |  | 0 | 0 | 1 | 0 | 0 | 12% | PD1 |
|  |  | Control ADC | 8 | 0 | 0 | 8 | 8 | 19.6 | 0.6 | 1.0 | p = 0.996 | p = 1.000 | 2.100±0.934 | p = 0.712 | 7.900±2.927 | 1.0 | p = 0.798 | 2.67(2.07,4.16) | 8 | 8 | 0 | 0 | 0 | 0 | 0 | 0 | 0% | PD1 |
|  |  | PVEK | 8 | 0 | 0 | 8 | 8 | 67.2 | 48.1 | 3.5 | p < 0.001 | p < 0.001 | 2.075±0.925 | p = 0.635 | 0.000±0.000 | 0.0 | p < 0.001 | -1.00(-1.00,-1.00) | 0 | 0 | 0 | 0 | 0 | 0 | 8 | 0 | 100% | MCR |
|  |  | Control ADC vs PVEK |  |  |  |  |  |  | 47.5 | 3.4 | p < 0.001 | p < 0.001 |  | p = 1.000 |  | 0.0 | p < 0.001 |  |  |  |  |  |  |  |  |  |  |  |
| MLLr-ALL | ALL-3 | Vehicle Control | 8 | 0 | 0 | 8 | 8 | 6.7 |  |  |  |  | 2.362±1.824 |  | 23.087±12.031 |  |  | 10.43(7.24,14.16) | 8 |  |  | 0 | 0 | 0 | 0 | 0 | 0% | PD1 |
|  |  | Control ADC | 8 | 0 | 0 | 8 | 8 | 8.1 | 1.4 | 1.2 | p = 0.798 | p = 0.835 | 2.450±1.433 | p = 0.792 | 25.812±12.581 | 1.1 | p = 1.000 | 10.72(6.86,15.09) | 8 | 8 | 0 | 0 | 0 | 0 | 0 | 0 | 0% | PD1 |
|  |  | PVEK | 8 |  |  | 8 | 1 | > 182 | > 175 | > 27.3 | p < 0.001 | p < 0.001 | 2.400±1.377 | p = 0.875 | 0.000±0.000 | 0.0 | p < 0.001 | -1.00(-1.00,-1.00) | 0 | 0 | 0 | 0 | 0 | 0 | 8 | 0 | 100% | MCR |
|  |  | Control ADC vs PVEK |  |  |  |  |  |  | > 174 | > 22.6 | p < 0.001 | p < 0.001 |  | p = 1.000 |  | 0.0 | p < 0.001 |  |  |  |  |  |  |  |  |  |  |  |
|  | MLL-1 | Vehicle Control | 8 | 0 | 0 | 8 | 8 | 12.2 |  |  |  |  | 3.812±3.553 |  | 14.500±7.364 |  |  | 3.35(2.27,4.81) | 8 |  |  | 0 | 0 | 0 | 0 | 0 | 0% | PD1 |
|  |  | Control ADC | 8 | 0 | 0 | 8 | 8 | 12.2 | 0 | 1.0 | p = 0.570 | p = 0.917 | 4.175±4.598 | p = 0.673 | 13.263±10.355 | 0.9 | p = 0.713 | 2.61(1.85,3.68) | 8 | 8 | 0 | 0 | 0 | 0 | 0 | 0 | 0% | PD1 |
|  |  | PVEK | 8 |  |  | 8 | 2 | > 168 | > 156 | > 13.8 | p < 0.001 | p < 0.001 | 3.675±2.587 | p = 0.958 | 0.000±0.000 | 0.0 | p < 0.001 | -1.00(-1.00,-1.00) | 0 | 0 | 0 | 0 | 0 | 0 | 8 | 0 | 100% | MCR |
|  |  | Control ADC vs PVEK |  |  |  |  |  |  | > 156 | > 13.8 | p < 0.001 | p < 0.001 |  | p = 0.916 |  | 0.0 | p < 0.001 |  |  |  |  |  |  |  |  |  |  |  |
|  | MLL-5 | Vehicle Control | 8 | 0 | 0 | 8 | 8 | 4.9 |  |  |  |  | 4.538±2.729 |  | 55.212±14.323 |  |  | 13.63(8.97,15.02) | 8 |  |  | 0 | 0 | 0 | 0 | 0 | 0% | PD1 |
|  |  | Control ADC | 8 | 0 | 0 | 8 | 8 | 5.1 | 0.2 | 1.1 | p = 0.685 | p = 0.403 | 4.013±2.365 | p = 0.753 | 51.337±8.972 | 0.9 | p = 0.382 | 11.57(9.26,22.54) | 8 | 8 | 0 | 0 | 0 | 0 | 0 | 0 | 0% | PD1 |
|  |  | PVEK | 8 | 0 | 0 | 8 | 8 | 15.8 | 10.9 | 3.3 | p < 0.001 | p < 0.001 | 5.125±3.828 | p = 0.959 | 11.412±9.940 | 0.2 | p < 0.001 | 0.81(0.33,1.86) | 6 | 1 | 5 | 0 | 1 | 1 | 0 | 0 | 25% | PD2 |
|  |  | Control ADC vs PVEK |  |  |  |  |  |  | 10.7 | 3.1 | p = 0.001 | p = 0.001 |  | p = 0.636 |  | 0.2 | p < 0.001 |  |  |  |  |  |  |  |  |  |  |  |

PDX, patient-derived xenograft; ADC, antibody-drug conjugate; PVEK, pivekimab sunirine; N, total number of mice entering experiment; Nd, number of mice experiencing toxic death; Nx, number of additional mice excluded from analysis; Na, number of mice in analysis; Nev, number of events, defined as huCD45 cells ≥ 25%; KM med, Kaplan-Meier estimate of median time-to-event (days); EFS, event-free survival; T-C, Treated minus Control; T/C, Treated divided by Control; minCD45, minimum %huCD45 at any timepoint after treatment initiation; Med resp, median response; Excl ORM, excluded from ORM analysis due to the %huCD45^+^ cells being <1% at the initiation of treatment.

**Supplementary Table S8. Results for the dose escalation efficacy study with PVEK.**

| **PDX** | **Treatment Group** | **EFS (days)** | **EFS T-C (days)** | **EFS T/C (days)** | **p-value** | **Score** | **ORM** |
| --- | --- | --- | --- | --- | --- | --- | --- |
| ALL-19 | Vehicle Control | 6.5 |  |  |  |  |  |
|  | PVEK 0.06 mg/kg | 23.9 | 17.4 | 3.5 | 0.0009 | 2 | PD2 |
|  | PVEK 0.12 mg/kg | 69.8 | 63.3 | 10.7 | 0.0002 | 10 | MCR |
|  | PVEK 0.24 mg/kg | 95.4 | 88.9 | 14.7 | 0.0004 | 10 | MCR |
| ALL-3 | Vehicle Control | 6.9 |  |  |  |  |  |
|  | PVEK 0.06 mg/kg | 240 | 233 | 34.8 | 0.0002 | 10 | MCR |
|  | PVEK 0.12 mg/kg | 274 | 267 | 39.7 | 0.0002 | 10 | MCR |
|  | PVEK 0.24 mg/kg | 449 | 311 | 65.1 | 0.0004 | 10 | MCR |

PVEK, pivekimab sunirine; PDX, patient-derived xenograft; EFS, event free survival; ORM, objective response measure

**Supplementary Table S9. Detailed summary table for the *in vivo* dose escalation study with PVEK.**

|  |  | **Animal Counts/Status** | | | | | **EFS Evaluation** | | | | **%huCD45 Evaluation** | | | | **Response Evaluation** | | | | | | | | | |
| --- | --- | --- | --- | --- | --- | --- | --- | --- | --- | --- | --- | --- | --- | --- | --- | --- | --- | --- | --- | --- | --- | --- | --- | --- |
| **PDX** | **Treatment Group** | **N** | **Nd** | **Nx** | **Na** | **Nev** | **KM med** | **EFS T-C** | **EFS T/C** | **p-value Gehan-Wilcoxon** | **CD45 d0 mean+/-SD** | **CD45 d0 p-value** | **minCD45 mean+/-SD** | **minCD45 p-value** | **PD** | **PD1** | **PD2** | **SD** | **PR** | **CR** | **MCR** | **Excl**  **ORM** | **Resp rate** | **Med resp** |
| ALL-19 | Vehicle Control | 8 | 0 | 0 | 8 | 8 | 6.46 |  |  |  | 4.74+/-4.4 |  | 29.38+/-14.69 |  | 6 | 0 | 0 | 0 | 1 | 0 | 0 | 1 | 14.29 | PD |
|  | PVEK (0.06 mg/kg) | 8 | 0 | 0 | 8 | 8 | 23.9 | 17.4 | 3.69 | 0.0009 | 4.75+/-5.58 | 0.9581 | 7.56+/-8.22 | 0.0101 | 0 | 2 | 4 | 0 | 0 | 1 | 1 | 0 | 25 | PD2 |
|  | PVEK (0.12 mg/kg) | 8 | 0 | 0 | 8 | 8 | 69.8 | 63.4 | 10.81 | 0.0002 | 3.56+/-2.67 | 0.7132 | 0+/-0 | 0.0004 | 0 | 0 | 0 | 0 | 0 | 0 | 8 | 0 | 100 | MCR |
|  | PVEK (0.24 mg/kg) | 8 | 0 | 1 | 7 | 7 | 95.4 | 88.9 | 14.76 | 0.0004 | 4.23+/-3.25 | 1 | 0+/-0 | 0.0008 | 0 | 0 | 0 | 0 | 0 | 0 | 6 | 1 | 100 | MCR |
|  | PVEK (0.06 mg/kg vs 0.12 mg/kg) |  |  |  |  |  |  | 46 | 2.93 | 0.0069 |  | 0.9163 |  | 0.0015 |  |  |  |  |  |  |  |  |  |  |
|  | PVEK (0.06 mg/kg vs 0.24 mg/kg) |  |  |  |  |  |  | 71.5 | 4 | 0.011 |  | 0.7723 |  | 0.0026 |  |  |  |  |  |  |  |  |  |  |
|  | PVEK (0.12 mg/kg vs 0.24 mg/kg) |  |  |  |  |  |  | 25.5 | 1.37 | 0.0084 |  | 0.6431 |  | 1 |  |  |  |  |  |  |  |  |  |  |
| ALL-3 | Vehicle Control | 8 | 0 | 0 | 8 | 8 | 6.89 |  |  |  | 2.92+/-1.12 |  | 26.14+/-5.26 |  | 8 | 0 | 0 | 0 | 0 | 0 | 0 | 0 | 0 | PD |
|  | PVEK (0.06 mg/kg) | 8 | 0 | 0 | 8 | 8 | 240 | 233 | 34.79 | 0.0002 | 2.79+/-1.43 | 0.9581 | 0+/-0 | 0.0004 | 0 | 0 | 0 | 0 | 0 | 0 | 7 | 1 | 100 | MCR |
|  | PVEK (0.12 mg/kg) | 8 | 0 | 3 | 5 | 3 | 274 | 267 | 39.79 | 0.0029 | 2.74+/-1.28 | 0.6084 | 0+/-0 | 0.0033 | 0 | 0 | 0 | 0 | 0 | 0 | 5 | 0 | 100 | MCR |
|  | PVEK (0.24 mg/kg) | 8 | 0 | 5 | 3 | 2 | 504 | 497 | 73.15 | 0.0209 | 1.53+/-0.87 | 0.0827 | 0+/-0 | 0.0178 | 0 | 0 | 0 | 0 | 0 | 0 | 3 | 0 | 100 | MCR |
|  | PVEK (0.06 mg/kg vs 0.12 mg/kg) |  |  |  |  |  |  | 34.5 | 1.14 | 0.0881 |  | 0.8835 |  | 1 |  |  |  |  |  |  |  |  |  |  |
|  | PVEK (0.06 mg/kg vs 0.24 mg/kg) |  |  |  |  |  |  | 264 | 2.1 | 0.0359 |  | 0.2196 |  | 1 |  |  |  |  |  |  |  |  |  |  |
|  | PVEK (0.12 mg/kg vs 0.24 mg/kg) |  |  |  |  |  |  | 230 | 1.84 | 0.461 |  | 0.136 |  | 1 |  |  |  |  |  |  |  |  |  |  |

PDX, patient-derived xenograft; PVEK, pivekimab sunirine; N, total number of mice entering experiment; Nd, number of mice experiencing toxic death; Nx, number of additional mice excluded from analysis; Na, number of mice in analysis; Nev, number of events, defined as huCD45 cells ≥ 25%; KM med, Kaplan-Meier estimate of median time-to-event (days); EFS, event-free survival; T-C, Treated minus Control; T/C, Treated divided by Control; CD45 d0, %huCD45 at day 0; minCD45, minimum %huCD45 at any timepoint after treatment initiation; Resp rate, response rate; Med resp, median response; Excl ORM, excluded from ORM analysis due to the %huCD45^+^ cells being <1% at the initiation of treatment.

**Supplementary Table S10. Comparison of *in vivo* PVEK efficacy across conventional, dose escalation, and SMT studies.**

|  |  |  | Conventional Study  (0.24 mg/kg PVEK) | | | Dose Escalation Study  (0.06 mg/kg PVEK) | | | Dose Escalation Study  (0.12 mg/kg PVEK) | | | Dose Escalation Study  (0.24 mg/kg PVEK) | | | SMT Study  (0.24 mg/kg PVEK) | | |
| --- | --- | --- | --- | --- | --- | --- | --- | --- | --- | --- | --- | --- | --- | --- | --- | --- | --- |
| Subtype | PDX | CD123 (FPKM) | EFS  (days) | ORM  Score | ORM | EFS  (days) | ORM  Score | ORM | EFS  (days) | ORM  Score | ORM | EFS  (days) | ORM  Score | ORM | EFS  (days) | ORM  Score | ORM |
| BCP-ALL | ALL-2 | 8.6 | 53.7 | 10 | MCR | - | - | - | - | - | - | - | - | - | 56.0 | 10 | MCR |
|  | ALL-7 | 8.8 | 76.4 | 10 | MCR | - | - | - | - | - | - | - | - | - | 82.5 | 10 | MCR |
|  | ALL-19 | 16.0 | 96.0 | 10 | MCR | 23.9 | 2 | PD2 | 69.8 | 10 | MCR | 95.4 | 10 | MCR | 90.2 | 10 | MCR |
| Ph+-ALL | ALL-4 | 13.3 | 26.7 | 2 | PD2 | - | - | - | - | - | - | - | - | - | 16.0 | 2 | PD2 |
|  | ALL-55 | 6.1 | 67.2 | 10 | MCR | - | - | - | - | - | - | - | - | - | 38.6 | 8 | CR |
| MLLr-ALL | ALL-3 | 11.2 | 232 | 10 | MCR | 240 | 10 | MCR | 274 | 10 | MCR | 449 | 10 | MCR | 379 | 10 | MCR |
|  | MLL-1 | 21.0 | 229 | 10 | MCR | - | - | - | - | - | - | - | - | - | * | 10 | MCR |
|  | MLL-5 | 51.4 | 15.8 | 2 | PD2 | - | - | - | - | - | - | - | - | - | 20.1 | 2 | PD2 |

PVEK, pivekimab sunirine; SMT, single mouse trial; PDX, patient-derived xenograft; FPKM, fragments per kilobase per million reads; EFS, event free survival; ORM, objective response measure

* humanely killed at day 62

**Supplementary Table S11. Comparison of the *ex vivo* DGN549 cytotoxicity assay and *in vivo* conventional study with PVEK**

|  |  | |  | | Cytotoxicity Assay | Conventional Study  (PVEK) | | |
| --- | --- | --- | --- | --- | --- | --- | --- | --- |
| Subtype | | PDX | | CD123 (FPKM) | DGN549  IC_50_ (nM) | EFS  (days) | ORM  Score | ORM |
| BCP-ALL | | ALL-2 | | 8.6 | 83.3 | 53.7 | 10 | MCR |
|  |  | ALL-7 | | 8.8 | 0.93 | 76.4 | 10 | MCR |
|  |  | ALL-19 | | 16.0 | 8.5 | 96.0 | 10 | MCR |
| Ph+-ALL | | ALL-4 | | 13.3 | 3.7 | 26.7 | 2 | PD2 |
|  |  | ALL-55 | | 6.1 | 4.7 | 67.2 | 10 | MCR |
| MLLr-ALL | | ALL-3 | | 11.2 | 44.2 | 232 | 10 | MCR |
|  |  | MLL-1 | | 21.0 | 91.2 | 229 | 10 | MCR |
|  |  | MLL-5 | | 51.4 | 3.7 | 15.8 | 2 | PD2 |

PVEK, pivekimab sunirine; PDX, patient-derived xenograft; FPKM, fragments per kilobase per million reads; EFS, event free survival; ORM, objective response measure
